# The interplay between social connection and compliance with COVID-19 preventive measures

**DOI:** 10.1093/eurpub/ckag023

**Published:** 2026-02-16

**Authors:** Kirsten A Verhaegen, Rana Charafeddine, Maaike Paredis, Valentien Taeldeman, Tom Loeys, Stefaan Demarest, Piet Bracke, Katrijn Delaruelle, Elise Braekman

**Affiliations:** Department of Epidemiology and Public Health, Sciensano, Brussels, Belgium; Department of Sociology, Faculty of Political and Social Sciences, UGent, Ghent, Belgium; Department of Epidemiology and Public Health, Sciensano, Brussels, Belgium; Department of Sociology, Faculty of Political and Social Sciences, UGent, Ghent, Belgium; Department of Sociology, Faculty of Political and Social Sciences, UGent, Ghent, Belgium; Department of Data Analysis, Faculty of Psychology and Educational Sciences, UGent, Ghent, Belgium; Department of Epidemiology and Public Health, Sciensano, Brussels, Belgium; Department of Sociology, Faculty of Political and Social Sciences, UGent, Ghent, Belgium; Department of Sociology, Faculty of Political and Social Sciences, UGent, Ghent, Belgium; Department of Epidemiology and Public Health, Sciensano, Brussels, Belgium

## Abstract

The COVID-19 pandemic required substantive preventive measures to stop the spread of the SARS-CoV-2 virus. As the effectiveness of these measures highly depended on public adherence, understanding the determinants of compliance with preventive measures is crucial. While some influencing factors have been identified already, the role of social connection is not fully understood. Interestingly, social connection might be reciprocally affected by compliance with preventive measures in a way that poses a trade-off between social connection and compliance. Therefore, the aim of the current study was to evaluate the bidirectional relationship between social connection and compliance with preventive measures. This was done using longitudinal data from six waves (April 2020–March 2021) of the Belgian online COVID-19 Health Surveys, of a cohort of 11 974 adults. Random-intercept cross-lagged panel modelling was used to test whether social connection positively predicted subsequent compliance, and whether compliance negatively predicted subsequent social connection. Social support and social satisfaction served as social connection indicators. Compliance was modelled per separate preventive measure. The results showed that social support and social satisfaction were positively associated with subsequent compliance with several measures. Some effects were particularly pronounced in the most stringent waves. In the other direction, compliance with social restriction negatively predicted subsequent social satisfaction. Through its relation with compliance, social connection can be relevant for tackling public health crises that require public response. Vice versa, our results stress social connection as an area of concern in such crises. Thus, sustainably fostering social connection could benefit future pandemic preparedness.

Key Points

- Social support positively predicted subsequent compliance with staying home measures, social restriction, and mask wearing.
- Social satisfaction positively predicted subsequent compliance with staying home measures, physical distancing and hygiene measures.
- In general, compliance did not predict subsequent social connection, with the exception of a negative effect of compliance with social restriction on subsequent social satisfaction.

## Introduction

During the COVID-19 pandemic, caused by the SARS-CoV-2 virus [1], substantial preventive measures were employed worldwide to limit the spread of the virus. Some of the most commonly used measures included hand washing, mask wearing, physical distancing (i.e. pertaining a distance of 1–2 m from people outside the household) [2], social restriction (i.e. limiting the number of social contacts outside the household), and quarantine in the case of (suspected) infection [3]. These measures were supported by scientific literature demonstrating their effectiveness in lowering transmission of the SARS-CoV-2 virus [4]. However, this largely depended on the extent to which the population adhered to the preventive measures [5].

Since compliance with preventive measures is crucial, researchers started to identify factors that influence compliant behaviour in the context of public health crises. Research on previous epidemics has suggested that compliance is related to factors such as age (i.e. higher compliance in older individuals) [6], gender (i.e. higher compliance in women) [7], fear [6], risk perception [6, 7], and trust in the government [8]. For the COVID-19 pandemic, the same factors were related to higher compliance [9–13]. However, the role of other factors has not yet been sufficiently clarified.

Among other factors, little is known about the role of social connection, even though many measures directly impacted social habits and were shaped by social influences [14, 15]. Social connection is an umbrella term that describes one’s social life in terms of structure, function, and quality [16]. The current study focused on functional social connection, which captures how one’s social network fulfils social needs. Common indicators are social support, social satisfaction, and loneliness. Considering the social nature of COVID-19 preventive measures, it is highly relevant to assess whether functional social connection and compliance with preventive measures affect each other.

While the literature on functional social connection and compliance in the context of COVID-19 is still scarce, early evidence showed negative correlations between loneliness and compliance [17, 18]. Social support findings were less consistent, with some finding no association between social support and compliance [19], and others finding different associations depending on the source of support (e.g. friends vs. family) [20, 21]. However, as these studies are cross-sectional, they cannot assess evolutions and interactions over time. A first longitudinal study on the topic found no cross-lagged effects of social connection on subsequent compliance (i.e. social connection at one timepoint did not predict compliance at the next timepoint) [22]. However, in women specifically, compliance with preventive measures at one timepoint was positively correlated with feelings of loneliness, depression, and unhappiness at the next timepoint. As this was, to our knowledge, the first and only longitudinal study on the topic, more studies on the bidirectional effects between social connection and compliance with COVID-19 preventive measures are needed. Particularly, there is no longitudinal research on the relation between positive indicators of functional social connection (e.g. social support) and compliance. This gap in the literature is important considering the observed protective effect of positive indicators, such as social support, on negative indicators such as loneliness [23].

To study the relationship between positive indicators of functional social connection (i.e. social support and social satisfaction) and compliance with COVID-19 preventive measures, we used longitudinal data from online questionnaires in the Belgian adult population. We hypothesized that compliance and functional social connection influence each other bidirectionally over time, resulting in two main hypotheses. First, we posed a positive relation between functional social connection and subsequent compliance with preventive measures, and particularly with physical distancing and social restriction (i.e. limiting the number of social contacts) (H1). We expected that this effect would be particularly present in the waves with more restricting preventive measures. Second, we hypothesized that compliance with preventive measures would be negatively related with subsequent social connection (H2). Again, we expected this to occur specifically in the more restricting phases of the pandemic.

## Methods

### Study design

**Figure 1.**
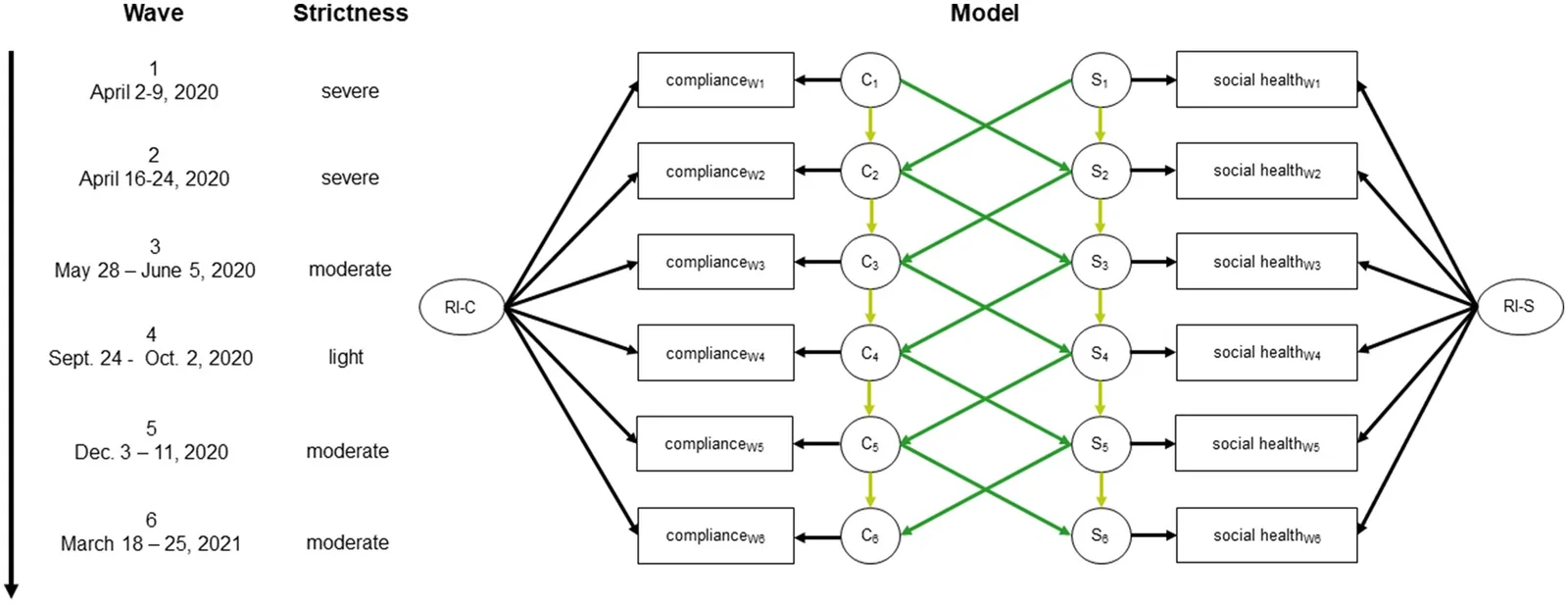
Overview of waves, strictness, and general model used for RI-CLPM analyses. RI = random intercept; C = compliance; W = wave; rectangular shapes = observed variables; round shapes = latent variables; dark green = cross-lagged effects; light green = auto-lagged effects; specific variables used for compliance: compliance with hygiene measures, physical distancing, staying home rules, social restriction, and face mask wearing; specific variables used for social connection: OSSS-3 social support, satisfaction with social contacts in the past 2 weeks. For clarity, covariances of the innovations and random intercepts are not shown in the figure. A general model formula can be found in Supplementary Material 3. For an in-depth discussion of RI-CLPM, see [30].

The COVID-19 Health Surveys were a series of 10 online surveys monitoring the impact of the COVID-19 pandemic on the health and well-being of the general Belgian population between April 2020 and March 2022 in a convenience sample. They could be completed in Dutch, French, or German (i.e. national languages). In the current study, we use data from the first six waves. Figure 1 shows the timing of each wave, along with the strictness levels of preventive measures as communicated by the Belgian national government, and is based on a more detailed methodological discussion of the surveys [24].

### Participants

The surveys were administered in adults (18+) living in Belgium. All surveys were approved by the ethical committee of the University Hospital of Ghent. For all surveys, a non-probability sampling approach was used. Participants were recruited using river sampling, snowball sampling, offline recruitment (press conferences and offline media news), and recruitment of previous participants (starting from the second wave). All participants provided informed consent to the terms and conditions of the survey following the General Data Protection Regulation (GDPR) [25] and the Declaration of Helsinki [26]. At the end of each survey, participants were asked for consent to data linking across survey waves, in which case they provided an e-mail address for this purpose. This linking was performed prior to and separately from the analyses to safeguard privacy. The current cohort consists of participants who agreed to linking and participated in at least 5 out of 10 surveys (*N* = 11 974). Sociodemographic details of the cohort can be found in Supplementary Material 1. While the cohort was not specifically aimed to be representative for the general population, an evaluation of its representativity can be found in Supplementary Material 1.

### Survey content

An overview of the relevant questions can be found in Supplementary Material 2.

#### Compliance with preventive measures

Participants were asked to what extent they complied with the specific measures enforced at that time, separately per measure (i.e. low, partial, or strict compliance). Most compliance questions were administered in the first six waves. Some preventive measures were imposed throughout the entire survey period (i.e. hygiene measures, physical distancing, and staying home measures), while other measures were imposed only from the third wave on (e.g. social restriction [i.e. prohibition to have social contacts with more than X people within a period, X varied over time], and mask wearing).

#### Social connection

Social support and social satisfaction served as social connection variables (see Supplementary Material 2). Social support was measured using the 3-item Oslo Social Support Scale (OSSS-3) [27]. More specifically, participants indicated how many people they can count on for support with personal problems, the extent to which people show interest and concern in them and access to practical help from neighbours. These questions formed a combined score between 3 and 14. A score between 3 and 8 indicated poor social support, while a score between 9 and 11 indicated moderate social support and scores above 11 indicated strong social support [27]. For social satisfaction, participants were asked to rate their satisfaction with their social contacts in the past 2 weeks on a scale from 1 (‘very satisfying’) to 4 (‘very unsatisfying’), which was later reversed for interpretation.

### Statistical analysis

Missing values were imputed with the highly flexible random forest imputation method [28], which predicts missing values through iterations of random forest estimation until convergence has been reached, resulting in a single completed dataset. It has been found to be a robust imputation method that preserves type 1 error rates [29]. The imputation was done using the missForest package in R [28], with 100 trees per random forest and a maximum of three imputation iterations. Using the wide-format dataset, each variable-wave combination was imputed across participants. Only observed variables with partially missing entries were imputed within each variable of each wave, and participants with incomplete wave participation were included.

The main analysis involved random-intercept cross-lagged panel modelling (RI-CLPM) [30^,^^31^], performed in R using the lavaan package [32]. Using this method, the bidirectional effects between two variables are tested over time, where one can assess auto-lagged effects (i.e. autocorrelation between *X_t_* and *X_t+1_*, light green arrows in Fig. 1) as well as cross-lagged effects (i.e. correlation between *X_t_* and *Y_t+1_*, dark green arrows in Fig. 1). This allowed us to assess whether social connection variables at one timepoint predict compliance at the next timepoint, and vice versa. Figure 1 shows the general model including the compliance and social connection variables, which were all interpreted continuously. Each compliance variable was modelled per separate preventive measure. Aside from global models, additional models were created where general wave strictness was included (see Fig. 1), by grouping cross-lagged effects based on strictness. All reported models showed good fit (i.e. all models had a comparative fit index and Tucker-Lewis index of 0.84 or higher and a root mean square of approximation of 0.08 or lower). All global effects and model fit measures are displayed in Supplementary Material 4.

## Results

### Descriptive analyses

The overall mean self-reported compliance score was 2.90 out of 3 (*SD* = 0.09), with the highest compliance for staying home measures (*M* = 2.96, *SD* = 0.19) and mask wearing (*M* = 2.98, *SD* = 0.17), and the lowest compliance for social restriction (*M* = 2.75, *SD* = 0.50). Over time, there was a mild but significant decline in compliance for all preventive measures (all *P* < .001), except for mask wearing (*P* = .494). A visualization of the data over time can be found in Supplementary Material 5.

Overall social support was moderately high (i.e. interpretation of a score between 9 and 11) [27], with an overall mean OSSS-3 score of 9.72 out of 14 (*SD* = 2.32). There was a small but statistically significant decline in social support over time (*P* < .001). When interpreted continuously, the overall mean social satisfaction score was 2.41 out of 4 (*SD* = 0.77), with 2 corresponding to ‘rather unsatisfying’ and 3 to ‘rather satisfying’.

### Random-intercept cross-lagged panel modelling

#### Compliance and social support

**Figure 2.**
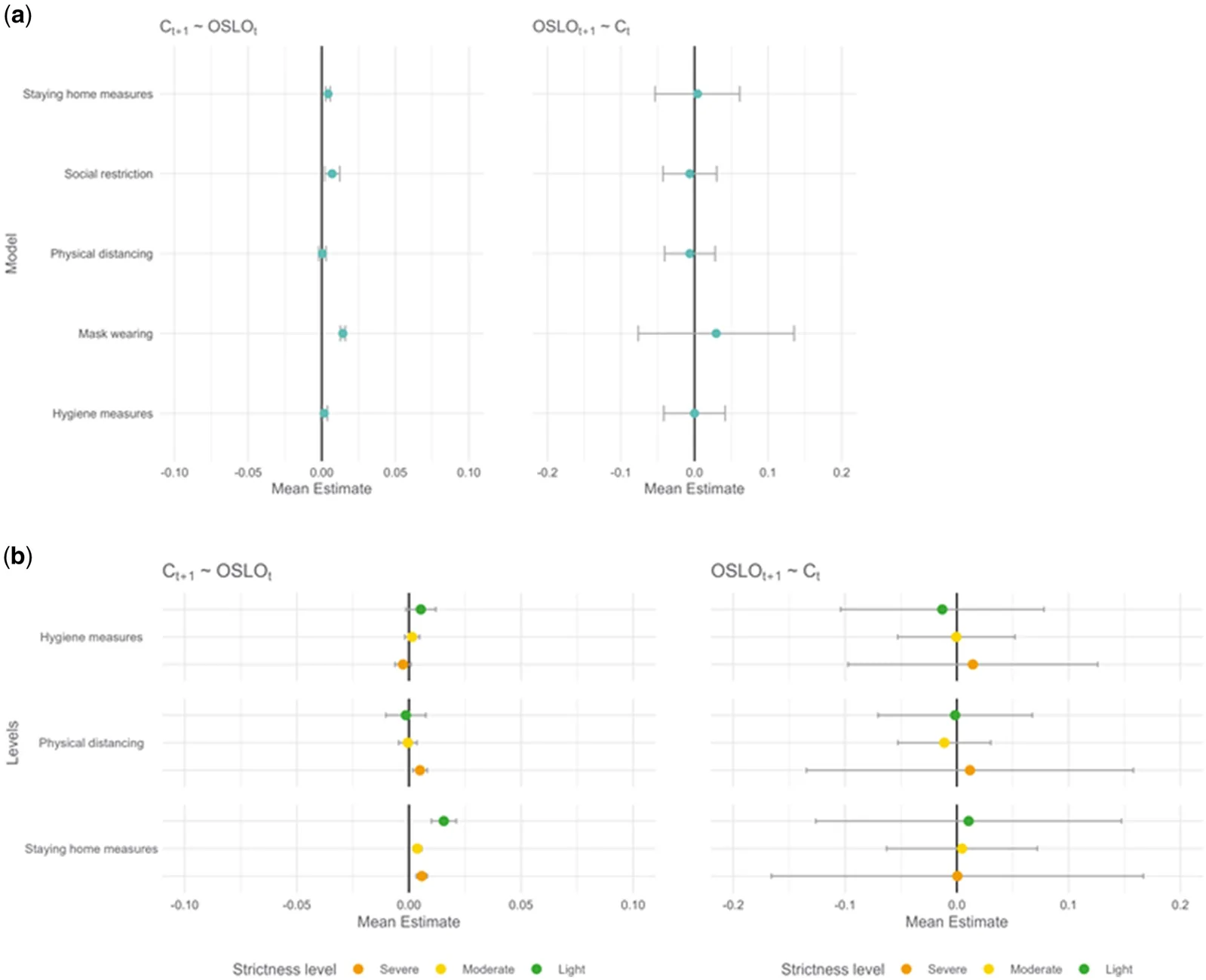
Cross-lagged effects between compliance and social support, in general (a) and per level of preventive measure strictness (b). Estimates reflect the mean cross-lagged effects (beta-weights) across waves, with positive values indicating a positive relation of one variable with the subsequent measurement of the other variable and negative values indicating a negative relation. All mean estimates are displayed with 95% confidence intervals. (a) Left panel: cross-lagged effects between social support scores and subsequent compliance. Right panel: cross-lagged effects between compliance and subsequent social support scores. (b) Social restriction and mask wearing are not shown because they were measured only in moderately strict waves. Left panel: cross-lagged effects of social support on subsequent compliance per level of strictness of enforced measures (waves with severe measures, moderate measures, and light measures). Right panel: cross-lagged effects of compliance on subsequent social support per level of strictness of enforced measures (waves with severe measures, moderate measures, and light measures).

In general, social support scores predicted subsequent compliance with staying home measures (*β* = .004, *P* < .001), social restriction (*β* = .007, *P* = .008), and mask wearing (*β* = .014, *P* < .001). None of the other cross-lagged effects were significant (all *P* > .171), including the effects of compliance on subsequent social support. Figure 2a shows all general cross-lagged effects. All auto-lagged effects were significant for both compliance and social support. This means that scores predicted subsequent scores on the same variable.

Analyses stratified by preventive measure strictness (Fig. 2b) showed a significant cross-lagged effect of social support on compliance with staying home measures for each strictness level. A cross-lagged effect of social support on subsequent compliance with physical distancing was present, specifically in the most strict waves (*β* = .077, *P* < .001). For each strictness level, all auto-lagged effects were significant. No such stratified analyses could be performed for social restriction measures and mask wearing, since these measures were only enforced later in the pandemic, when strictness levels were consistently moderate.

#### Compliance and social satisfaction

**Figure 3.**
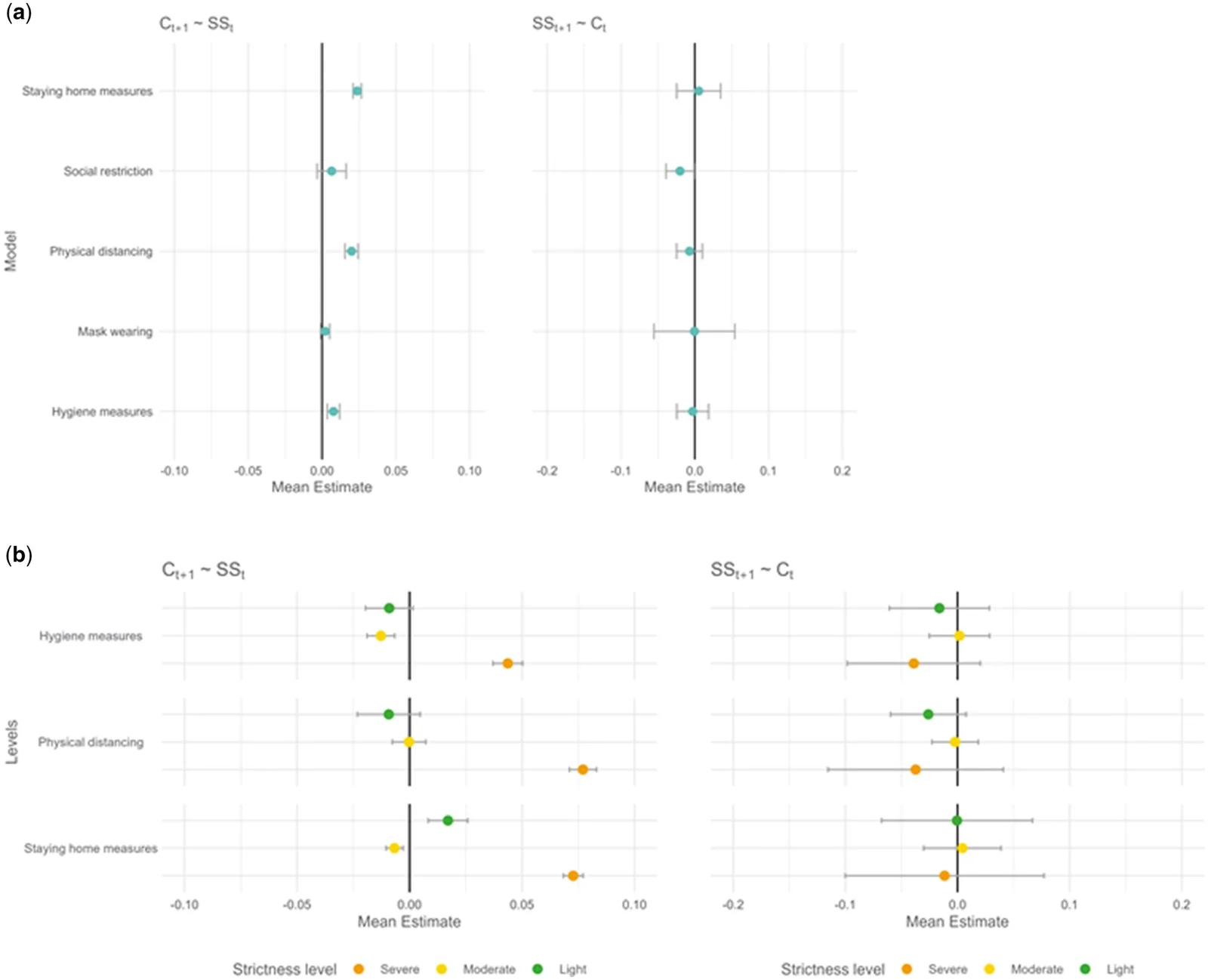
Cross-lagged effects between compliance and social satisfaction, in general (a) and per level of preventive measure strictness (b). Estimates reflect the mean cross-lagged effects (beta-weights) across waves, with positive values indicating a positive relation of one variable with the subsequent measurement of the other variable and negative values indicating a negative relation. All mean estimates are displayed with 95% confidence intervals. (a) Left panel: cross-lagged effects of social satisfaction on subsequent compliance. Right panel: cross-lagged effects of compliance on subsequent social satisfaction. (b) Social restriction and mask wearing are not shown because they were measured only in moderately strict waves. Left panel: cross-lagged effects of social satisfaction on subsequent compliance per level of strictness of enforced measures (waves with severe measures, moderate measures, and light measures). Right panel: cross-lagged effects of compliance on subsequent social satisfaction per level of strictness of enforced measures (waves with severe measures, moderate measures, and light measures).

In general, satisfaction with social contacts predicted subsequent compliance with several measures. More specifically, there was a significant positive cross-lagged effect of social satisfaction on subsequent compliance with staying home measures (*β* = .024, *P* < .001), physical distancing (*β* = .020, *P* < .001), and hygiene measures (*β* = .008, *P* < .001). The effects of social satisfaction on compliance with social restriction and mask wearing were not significant (all *P* > .181). In the other direction, there was a significant negative cross-lagged effect of compliance with social restriction on subsequent social satisfaction (*β* = −.020, *P* = .039). None of the other cross-lagged effects were significant (all *P* > .423). All cross-lagged effects are displayed in Fig. 3a. All auto-lagged effects were significant.

Analyses stratified by preventive measure strictness indicated that the significant positive cross-lagged effects of social satisfaction on compliance were particularly present in those periods of the pandemic when enforced preventive measures were most strict. This was the case for compliance with hygiene measures (*β* = .044, *P* < .001), physical distancing (*β* = .077, *P* < .001), and staying home measures (*β* = .73, *P* < .001). However, for the moderate strictness level, there was a negative cross-lagged effect of social satisfaction on compliance with hygiene measures (*β* = −.013, *P* < .001) and staying home measures (*β* = −.017, *P* < .001). For the lightest strictness level, the cross-lagged effect of social satisfaction on compliance with staying home measures was significant and positive (*β* = .017, *P* < .001). For each strictness level, all auto-lagged effects were also significant. Again, no such stratified analyses could be performed for social restriction measures and mask wearing. The results of the strictness-based results are shown in Fig. 3b.

#### Post-hoc analyses

Post-hoc analyses explored why social support and social satisfaction were positively related to subsequent compliance with different measures. Plausibly, both variables tap into different aspects of social connection, thereby relating to compliance differently. Social support emphasizes a practical form of social connection, whereas social satisfaction has a stronger emotional emphasis. However, the OSSS-3 contains one question that reflects a more emotional, rather than practical kind of support. Post-hoc RI-CLPM analyses with only this second item showed significant positive cross-lagged effects on subsequent compliance with all preventive measures (all *P* < .013), and no significant cross-lagged effects in the other direction. In contrast, the analysis with the other separate items showed few significant cross-lagged effects. Having more close people to count on negatively predicted subsequent compliance with physical distancing (*β* = −.010, *P* < .001). There also was a negative effect of having easy access to help from neighbours on subsequent compliance with hygiene measures (*β* = −.006, *P* = .010). Additionally, there was a positive effect of having easy access to help from neighbours on subsequent compliance with mask wearing (*β* = .018, *P* < .001).

## Discussion

In line with our first hypothesis (H1), we observed that higher levels of social support and social satisfaction were related to higher subsequent compliance. For social support, positive cross-lagged effects were found on subsequent compliance with staying home measures, social restriction, and mask wearing. For social satisfaction, effects were observed for compliance with staying home measures, physical distancing, and hygiene measures. When combined, the presence of several positive cross-lagged effects can be seen as support for H1. However, it was unclear why social support and social satisfaction predict compliance with some but not all measures, and why both variables predict compliance with different measures. Post-hoc analyses separating the OSSS-3 items showed consistent cross-lagged effects of the second, more emotional item on subsequent compliance with all measures, but no such results for the other, more practical OSSS-3 items. However, there might be alternative explanations, and still, this does not explain why social satisfaction did not predict compliance with some, but not all measures. Future research could explore this and other explanations, among which the influence of potential ceiling effects. In addition, it is important to also consider the significant auto-lagged effects, which had larger effect sizes than the cross-lagged effects. This means that there is a relatively high degree of within-person stability of social connection and compliance over time.

The observed relation between social connection and subsequent compliance is in line with previous studies with other social connection indicators, showing lower compliance with preventive measures in those suffering from loneliness [18] or decreased pro-social behaviour after induced feelings of loneliness [33]. However, in a RI-CLPM study by Wright et al. [22], no significant cross-lagged effects of social connection on compliance were found. International differences in policy stringency might have influenced the relationship between social connection and compliance, as well as the way social connection and compliance are perceived and measured [34,35]. Indeed, in contrast with our global results, when separating cross-lagged effects per level of strictness, we observed negative cross-lagged effects of social satisfaction on compliance with staying home measures and hygiene measures in moderately strict waves only. One possible explanation could be that compliance was taken less seriously in moderate waves, in which individuals might have felt there was room for prioritization of social connection. This could have led to an initial increase in social satisfaction, followed by lower compliance. However, following this rationale, we would expect to observe this in the light waves too.

In the other direction, only compliance with social restriction measures significantly predicted subsequent social satisfaction. More specifically, higher compliance with social restriction was related to lower subsequent social satisfaction. This is in line with our second hypothesis (H2), and literature on the adverse psychological effects of quarantines and solitary confinement [36, 37]. However, we found no other significant cross-lagged effects of compliance on social satisfaction or social support. This suggests that stricter measures can be implemented in acute crises without immediate negative effects. However, such influences might develop more slowly than the cross-lagged effects that were tested in our study. Therefore, future studies should examine longer-term and accumulating influences, thereby also including potential protective factors such as digital connection.

Taken together, our results indicate that social connection can be relevant during public health crises such as the COVID-19 pandemic. Both social support and social satisfaction predicted subsequent compliance, mostly when using more emotion-focused measures of social connection. At the same time, complying with social restriction measures predicted lower social satisfaction. These findings argue for increased attention for social connection during and in anticipation of crises, as preventive measures can have consequences for social connection and feeling socially healthy can help people to do their part. In this way, the current study contributes to the existing knowledge on the determinants of preventive behaviour in a way that can help improve responses to future pandemics. By using longitudinal data, we moved beyond cross-sectional associations and gained deeper understanding of how social connection and compliance affect each other, while considering the strictness of measures.

However, of course, this study also has some limitations. First, as compliance was rated on a three-point scale and overall mean compliance scores were high (i.e. potential ceiling effect), our measurement of compliance was suboptimal for continuous interpretation in light of RI-CLPM. While model diagnostics showed no issues and such continuous interpretation has been argued to be valid with a big sample size [38], this still might have violated assumptions in a way that could have impacted our results. As a robustness test, we refitted the models using Bayesian estimation, with the blavaan package [39] (see Supplementary Material 6). It has been shown that Bayesian estimation is more robust to non-normality, is suited for categorical structural equation modelling, and shows better convergence than alternative estimators when the number of categories is small [40]. The results related to social satisfaction were largely confirmed, whereas the results related to social support were not. Follow-up research could explore these differences with a more optimal compliance measure. Second, we used self-reported compliance measures, which could differ from actual compliance, as it has already been observed that compliance with mask wearing tends to be overreported [14]. Together with a ceiling effect introduced by the narrow scale, social desirability may have led to high overall compliance scores that remained high, despite a significant decline over time. Considering the gravity with which the population was urged to adopt the preventive measures, social desirability possibly encouraged overreporting of compliance, despite the anonymous nature of the survey. The use of a three-point scale might also have contributed to overreporting, as nuanced response options lacked. Third, due to the sampling method and recruitment strategy, there might be a sampling bias. As there was a mass-communicated call for participation in COVID-19 surveys, it is plausible that individuals who already felt motivated towards action against COVID-19 were also more motivated to fill in a survey on the topic, and to comply with preventive measures. This might be another explanation for the observed high compliance levels. The suboptimal representativity of the cohort also points towards potential bias. As there were, however, no alternatives available, survey infrastructure could be improved in anticipation of future crises. Fourth, the main models do not test whether effects are homogeneous across sociodemographic subgroups. Therefore, the main analyses were repeated in stratified analyses (see more detailed report in Supplementary Material 7), which highlight a few points. First, generally, the results of the stratified analyses are in line with the main analyses. There are significant cross-lagged effects of social connection on subsequent compliance with several measures, but almost no cross-lagged effects in the other direction. Second, some observed cross-lagged effects of social connection on subsequent compliance were nuanced, when the effect was present only in particular subgroups. For example, the positive effect of social support on compliance with social restriction was particularly found in those groups that are already known to be more vulnerable for poor social connection (i.e. those without tertiary education, those living alone, women and those aged 65 years or older). Additionally, several associations differed in function of housing situation (i.e. living alone vs. not), pointing towards differences in perception and consequences of preventive measures. Finally, some diverging results were found for the effect of social connection on subsequent compliance. This stresses the importance of the personal and social context. Follow-up research could pinpoint the circumstances that influence this relationship.

Despite these limitations, the current study still made a crucial contribution to the literature on social connection and (COVID-19) preventive behaviour. Our results highlight the public health interest in enhancing social connection in anticipation of crises. Helping those with poor social connection will not only help them individually but will also benefit society by increasing the degree of compliance with necessary preventive measures. This also underscores the importance of preventive measures that are not unnecessarily strict. Taken together, these insights can inform responses to future pandemics or other large-scale crises that demand social sacrifices.

## Supplementary Material

ckag023_Supplementary_Data

## Data Availability

Access to the data reported in this article can be granted on reasonable request to HIS@sciensano.be.

## References

[1] Hu B , Guo H, Zhou P et al Characteristics of SARS-CoV-2 and COVID-19. Nat Rev Microbiol 2021;19:141–54. 10.1038/s41579-020-00459-7 33024307 PMC7537588

[2] Wasserman D , van der Gaag R, Wise J. The term “physical distancing” is recommended rather than “social distancing” during the COVID-19 pandemic for reducing feelings of rejection among people with mental health problems. Eur Psychiatry 2020;63:e52. 10.1192/j.eurpsy.2020.60 32475365 PMC7287304

[3] World Health Organization. Advice for the Public: Coronavirus Disease (COVID-19). Geneva: World Health Organization. https://www.who.int/emergencies/diseases/novel-coronavirus-2019/advice-for-public. Date accessed May 6 2025.

[4] Talic S , Shah S, Wild H et al Effectiveness of public health measures in reducing the incidence of covid-19, SARS-CoV-2 transmission, and covid-19 mortality: systematic review and meta-analysis. BMJ 2021;375:e068302, 10.1136/bmj-2021-068302 34789505 PMC9423125

[5] Chang SL , Harding N, Zachreson C et al Modelling transmission and control of the COVID-19 pandemic in Australia. Nat Commun 2020;11:5710. 10.1038/s41467-020-19393-6 33177507 PMC7659014

[6] Bults M , Beaujean DJ, de Zwart O et al Perceived risk, anxiety, and behavioural responses of the general public during the early phase of the influenza A (H1N1) pandemic in The Netherlands: results of three consecutive online surveys. BMC Public Health 2011;11:2. 10.1186/1471-2458-11-2 21199571 PMC3091536

[7] Bish A , Michie S. Demographic and attitudinal determinants of protective behaviours during a pandemic: a review. Br J Health Psychol 2010;15:797–824. 10.1348/135910710X485826 20109274 PMC7185452

[8] van der Weerd W , Timmermans DR, Beaujean DJ et al Monitoring the level of government trust, risk perception and intention of the general public to adopt protective measures during the influenza A (H1N1) pandemic in The Netherlands. BMC Public Health 2011;11:575. 10.1186/1471-2458-11-575 21771296 PMC3152536

[9] Bargain O , Aminjonov U. Trust and compliance to public health policies in times of COVID-19. J Public Econ 2020;192:104316. 10.1016/j.jpubeco.2020.104316 33162621 PMC7598751

[10] Brouard S , Vasilopoulos P, Becher M. Sociodemographic and psychological correlates of compliance with the COVID-19 public health measures in France. Can J Pol Sci 2020;53:253–8. 10.1017/S0008423920000335

[11] Kehr HM , Bakaç C, Jais M et al The role of Death-Anxiety-Induced fear of COVID-19 in compliance with and acceptance of Government-Issued COVID-19 regulations. Front Psychol 2022;13:881603. 10.3389/fpsyg.2022.881603 35586230 PMC9108415

[12] Lin T , Harris EA, Heemskerk A et al A multi-national test on self-reported compliance with COVID-19 public health measures: the role of individual age and gender demographics and countries’ developmental status. Soc Sci Med 2021;286:114335. 10.1016/j.socscimed.2021.114335 34450390 PMC8378016

[13] Lin T , Heemskerk A, Harris EA et al Risk perception and conspiracy theory endorsement predict compliance with COVID-19 public health measures. Br J Psychol 2023;114:282–93. 10.1111/bjop.12613 36414246 PMC10046644

[14] Woodcock A , Schultz PW. The role of conformity in mask-wearing during COVID-19. PLoS One 2021;16:e0261321. 10.1371/journal.pone.0261321 34919589 PMC8682910

[15] Grundmann F , Epstude K, Scheibe S. Face masks reduce emotion-recognition accuracy and perceived closeness. PloS One 2021;16:e0249792, 10.1371/journal.pone.0249792 33891614 PMC8064590

[16] Holt-Lunstad J. Social connection as a public health issue: the evidence and a systemic framework for prioritizing the “social” in social determinants of health. Annu Rev Public Health 2022;43:193–213. 10.1146/annurev-publhealth-052020-110732 35021021

[17] Okruszek Ł , Aniszewska-Stańczuk A, Piejka A et al Safe but lonely? Loneliness, anxiety, and depression symptoms and COVID-19. Front Psychol 2020;11:579181. 10.3389/fpsyg.2020.579181 33343454 PMC7747668

[18] Stickley A , Matsubayashi T, Ueda M. Loneliness and COVID-19 preventive behaviours among japanese adults. J Public Health (Oxf) 2021;43:53–60. 10.1093/pubmed/fdaa151 32880635 PMC7499629

[19] Shrestha N , Koju R, K.C D et al Perceived social support and compliance on stay-at-home order during COVID-19 emergency in Nepal: an evidence from web-based cross-sectional study. BMC Public Health 2023;23:535. 10.1186/s12889-023-15396-2 36944968 PMC10028774

[20] Paykani T , Zimet GD, Esmaeili R et al Perceived social support and compliance with stay-at-home orders during the COVID-19 outbreak: evidence from Iran. BMC Public Health 2020;20:1650. 10.1186/s12889-020-09759-2 33148209 PMC7609821

[21] Hills S , Eraso Y. Factors associated with non-adherence to social distancing rules during the COVID-19 pandemic: a logistic regression analysis. BMC Public Health 2021;21:352. 10.1186/s12889-021-10379-7 33581734 PMC7881344

[22] Wright L , Steptoe A, Fancourt D. Predictors of self-reported adherence to COVID-19 guidelines. A longitudinal observational study of 51,600 UK adults. Lancet Reg Health Eur 2021;4:100061. 33997831 10.1016/j.lanepe.2021.100061 PMC7907734

[23] Bareket‐Bojmel L , Shahar G, Abu‐Kaf S et al Perceived social support, loneliness, and hope during the COVID‐19 pandemic: testing a mediating model in the UK, USA, and Israel. British J Clinic Psychol 2021;60:133–48. 10.1111/bjc.12285 PMC8013849 33624294

[24] Braekman E, , Charafeddine R, , Berete F et al Data collection in pandemic times: The case of the belgian COVID-19 health surveys. Arch Public Health 2023;81. 10.1186/s13690-023-01135-x PMC10318627 37403166

[25] European Parliament, Council of the European Union, Regulation (EU) 2016/679 of the European parliament and of the council of 27 April 2016 on the protection of natural persons with regard to the processing of personal data and on the free movement of such data (General Data Protection Regulation). Off J Eur Union 2016;L119:1–88.

[26] World Medical Association. World Medical Association Declaration of Helsinki: ethical principles for medical research involving human participants. JAMA 2025;333:71–4. 39425955 10.1001/jama.2024.21972

[27] Kocalevent R-D , Berg L, Beutel ME et al Social support in the general population: standardization of the Oslo Social Support Scale (OSSS-3). BMC Psychol 2018;6:31. 10.1186/s40359-018-0249-9 30016997 PMC6050647

[28] Stekhoven DJ , Bühlmann P. MissForest—non-parametric missing value imputation for mixed-type data. Bioinformatics 2012;28:112–8. 10.1093/bioinformatics/btr597 22039212

[29] Guo C-Y , Yang Y-C, Chen Y-H. The optimal machine learning-based missing data imputation for the cox proportional hazard model. Front Public Health 2021;9:680054. 10.3389/fpubh.2021.680054 34291028 PMC8289437

[30] Hamaker EL , Kuiper RM, Grasman RP. A critique of the cross-lagged panel model. Psychol Methods 2015;20:102–16. 10.1037/a0038889 25822208

[31] Mulder JD, , Hamaker EL. Three extensions of the random intercept cross-lagged panel model. Structural Equation Modeling: A Multidisciplinary Journal 2021;28:638–48. 10.1080/10705511.2020.1784738

[32] Rosseel Y. Lavaan : an R package for structural equation modeling. J Stat Soft 2012;48:3–36. 10.18637/jss.v048.i02

[33] Yin M , Lee E-J. Exposure to loneliness cues reduces prosocial behavior: evidence from N400 and P300. Front Psychol 2023;14:1094652. 10.3389/fpsyg.2023.1094652 37138978 PMC10150042

[34] Al-Zubaidy N , Fernandez Crespo R, Jones S et al Exploring the relationship between government stringency and preventative social behaviours during the COVID-19 pandemic in the United Kingdom. Health Informatics J 2023;29:14604582231215867. 10.1177/14604582231215867 37982397

[35] Waterschoot J , Morbée S, Bergh OVD et al How the stringency of the COVID-19 restrictions influences motivation for adherence and well-being: the critical role of proportionality. Int J Health Policy Manag 2023;12:8021. 10.34172/ijhpm.2023.8021 38618783 PMC10699813

[36] Brooks SK , Webster RK, Smith LE et al The psychological impact of quarantine and how to reduce it: rapid review of the evidence. Lancet 2020;395:912–20. 10.1016/S0140-6736(20)30460-8 32112714 PMC7158942

[37] Haney C. The psychological effects of solitary confinement: a systematic critique. Crime Justice 2018;47:365–416. 10.1086/696041

[38] Norman G. Likert scales, levels of measurement and the “laws” of statistics. Adv Health Sci Educ Theory Pract 2010;15:625–32. 10.1007/s10459-010-9222-y 20146096

[39] Merkle EC , Rosseel Y. blavaan : Bayesian structural equation models via parameter expansion. J Stat Soft 2018;85:1–30. 10.18637/jss.v085.i04

[40] Liang X , Yang Y. An evaluation of WLSMV and Bayesian methods for confirmatory factor analysis with categorical indicators. IJQRE 2014;2:17.

