# Supplementary material for: The interplay between social connection and compliance with COVID-19 preventive measures
Source: Eur J Public Health. 2026 Feb 16;36(3):ckag023. doi: 10.1093/eurpub/ckag023 (PMC13230491; doi:10.1093/eurpub/ckag023)
Supplement: ckag023_Supplementary_Data [file ckag023_supplementary_data.zip › ejph-2025-05-om-0369-File005.docx]

**Supplementary Material 1**

**Table 1**

*Socio-demographic details of the current cohort (N = 11 974)*

|  | *N* | % of cohort |
| --- | --- | --- |
| **Sex** |  |  |
| Men | 4355 | 36.37 |
| Women | 7619 | 63.63 |
| **Age** |  |  |
| 18-24 years | 674 | 5.63 |
| 25-34 years | 1015 | 8.48 |
| 35-44 years | 1906 | 15.92 |
| 45-54 years | 2353 | 19.65 |
| 55-65 years | 3010 | 25.14 |
| 65+ years | 3016 | 25.19 |
| **Level of education** |  |  |
| No higher education | 2954 | 24.67 |
| Higher education | 8639 | 72.15 |
| Not disclosed | 381 | 3.18 |
| **Household situation** |  |  |
| Living alone | 2940 | 24.55 |
| Not living alone | 9034 | 75.45 |

*Note*. Respondents participated in at least five out of ten waves of the COVID-19 Health Surveys.

*Cohort representativity*

The cohort’s representativity for the Belgian population was evaluated in terms of sex, household type (living alone vs. not living alone), education (higher education vs. no higher education) and age group. Figure 6 shows the difference between (unweighted) cohort and population percentages for these sociodemographic variables. This shows a significant overrepresentation of women (+12.46%), of people not living alone (+11.49%) and of people with higher education (+5.05%) (all *p* < .001). Furthermore, younger age groups were underrepresented (i.e., 18-to-24 year-olds and 25-to-34 year-olds), whereas older age groups, and particularly the 55-to-64 year-olds, were overrepresented (*p* < .001).

**Figure 4**

*
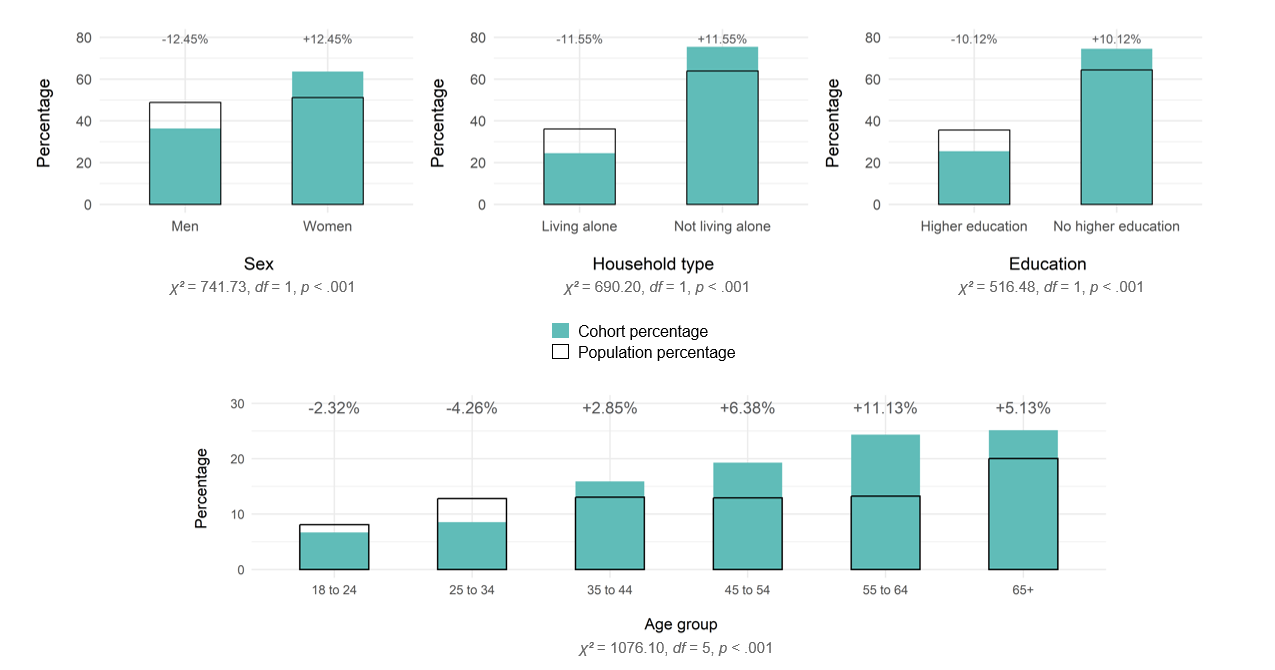
Cohort representativity for the Belgian population*

*Note.* Cohort percentages of sociodemographic groups in comparison to population percentages (obtained from Statbel).

**Supplementary Material 2**

**Compliance with preventive measures**

To what extent do you respect the following measures since their introduction?

|  | Strict respect | Partial respect | Low respect |
| --- | --- | --- | --- |
| Hygiene measures (Washing hands regularly, coughing in elbow, …) | o | o | o |
| Maintain a distance of at least 1.5 m from people (outside the people you live with) | o | o | o |
| Stay at home (except for work, medical care, supermarkets, pharmacy, newspapers, bank, post, and to help someone in need) | o | o | o |
| The social bubble measure: maximal four/ten (depending on timing) other people can be invited to your home. These are always the same four people and you can only visit these people in their home(s) as well. | o | o | o |
| Maintain a distance of at least 1.5 m when you invite people from your social bubble or visit them | o | o | o |
| Cover mouth and nose with a face mask, scarf or bandana on public transport/in public spaces | o | o | o |
| Cover mouth and nose with a face mask, scarf or bandana on other places where a distance of at least 1.5 m from other people cannot be guaranteed | o | o | o |
| Pay extra attention when meeting people from a high-risk group (e.g. people aged 65 and older, people with heart, lung or kidney diseases...) | o | o | o |

*Note.* Measured in waves 1 to 6, and 9 (with differences between specific statements)

**Social connection**

*Question 1*

In the last two weeks, how would you judge your social contacts?

- Really satisfying
- Rather satisfying
- Rather unsatisfying
- Really unsatisfying

Note: measured in waves 1 to 10

*Question 2 (OSSS-3 item 1)*

How many people are so close to you that you can count on them if you have serious personal problems?

- None
- 1 or 2
- 3 to 5
- 6 or more

Note: measured in waves 1 to 10

*Question 3 (OSSS-3 item 2)*

How much interest and concern do people show in what you are doing?

- A lot of concern and interest
- Some concern and interest
- Uncertain
- Little concern and interest
- No concern and interest

Note: measured in waves 1 to 10

*Question 4 (OSSS-3 item 3)*

How easy is it to get practical help from neighbors if you should need it?

- Very easy
- Easy
- Possible
- Difficult
- Very difficult

Note: measured in waves 1 to 10

**Supplementary Material 3**

**General model formula**

This formula was based on R code provided by Mulder et al. [31]. The variable names in this formula were generalized to show the overall formula used for specific compliance and social connection variables. The model is visualized in Figure 1 (see Methods). The lagged effects were labelled in order to calculate global effects (i.e., across waves), with a = auto-lagged effect of the compliance variable, b = cross-lagged effect of the social connection variable on the subsequent measurement of the compliance variable, c = cross-lagged effect of the compliance variable on the subsequent measurement of the social connection variable and d = auto-lagged effect of the social connection variable.

'

# Create between components (random intercepts)

RIx =~ 1*compliance_T1 + 1* compliance_T2 + 1* compliance_T3 + 1* compliance_T4 + 1* compliance_T5 + 1* compliance_T6 + 1* compliance_T9

RIy =~ 1*socialhealth_T1 + 1* socialhealth _T2 + 1* socialhealth _T3 + 1* socialhealth_T4 + 1* socialhealth _T5 + 1* socialhealth _T6 + 1* socialhealth _T9

# Create within-person centered variables

wx1 =~ 1*compliance_T1

wx2 =~ 1*compliance_T2

wx3 =~ 1*compliance_T3

wx4 =~ 1*compliance_T4

wx5 =~ 1*compliance_T5

wx6 =~ 1*compliance_T6

wx9 =~ 1*compliance_T9

wy1 =~ 1*socialhealth_T1

wy2 =~ 1*socialhealth_T2

wy3 =~ 1*socialhealth_T3

wy4 =~ 1*socialhealth_T4

wy5 =~ 1*socialhealth_T5

wy6 =~ 1*socialhealth_T6

wy9 =~ 1*socialhealth_T9

# Estimate lagged effects between within-person centered variables

wx2 ~ a*wx1 + b*wy1

wy2 ~ c*wx1 + d*wy1

wx3 ~ a*wx2 + b*wy2

wy3 ~ c*wx2 + d*wy2

wx4 ~ a*wx3 + b*wy3

wy4 ~ c*wx3 + d*wy3

wx5 ~ a*wx4 + b*wy4

wy5 ~ c*wx4 + d*wy4

wx6 ~ a*wx5 + b*wy5

wy6 ~ c*wx5 + d*wy5

# Estimate covariance between within-person centered variables at first wave

wx1 ~~ wy1

# Estimate covariances between residuals of within-person centered variables

# (i.e., innovations)

wx2 ~~ wy2

wx3 ~~ wy3

wx4 ~~ wy4

wx5 ~~ wy5

wx6 ~~ wy6

wx9 ~~ wy9

# Estimate variance and covariance of random intercepts

RIx ~~ RIx

RIy ~~ RIy

RIx ~~ RIy

# Estimate (residual) variance of within-person centered variables

wx1 ~~ wx1

wy1 ~~ wy1

wx2 ~~ wx2

wy2 ~~ wy2

wx3 ~~ wx3

wy3 ~~ wy3

wx4 ~~ wx4

wy4 ~~ wy4

wx5 ~~ wx5

wy5 ~~ wy5

wx6 ~~ wx6

wy6 ~~ wy6

wx9 ~~ wx9

wy9 ~~ wy9

'

**Supplementary Material 4**

**Table 2**

*Summarized results of RI-CLPM model for compliance with hygiene measures and social support*

| **Effect** | **Mean estimate** | **Mean SE** | **Z value** | **95% CI Lower** | **95% CI Upper** | ***p*** |
| --- | --- | --- | --- | --- | --- | --- |
| C_t_ 🡪 C_t+1_ | 0.182 | 0.00577 | 31.6 | 0.171 | 0.193 | < .0001 |
| S_t_ 🡪 C_t+1_ | 0.00161 | 0.00117 | 1.37 | -0.000692 | 0.00391 | .1707 |
| C_t_ 🡪 S_t+1_ | -0.0000473 | 0.0213 | -0.00223 | -0.0417 | 0.0416 | .9982 |
| S_t_ 🡪 S_t+1_ | 0.165 | 0.00552 | 29.8 | 0.154 | 0.176 | < .0001 |

*Note*. C = compliance with hygiene measures, S = social support

**Table 3**

*Summarized results of RI-CLPM model for compliance with hygiene measures and social satisfaction*

| **Effect** | **Mean estimate** | **Mean SE** | **Z value** | **95% CI Lower** | **95% CI Upper** | ***p*** |
| --- | --- | --- | --- | --- | --- | --- |
| C_t_ 🡪 C_t+1_ | 0.182 | 0.00577 | 31.60 | 0.171 | 0.193 | < 0.001 |
| S_t_ 🡪 C_t+1_ | 0.00772 | 0.00213 | 3.63 | 0.00355 | 0.0119 | < .001 |
| C_t_ 🡪 S_t+1_ | -0.00286 | 0.0110 | -0.260 | -0.0244 | 0.0187 | 0.7945 |
| S_t_ 🡪 S_t+1_ | 0.142 | 0.00524 | 27.10 | 0.132 | 0.153 | < 0.001 |

*Note*. C = compliance with hygiene measures, S = social satisfaction

**Table 4**

*Summarized results of RI-CLPM model for compliance with physical distancing and social support*

| **Effect** | **Mean estimate** | **Mean SE** | **Z value** | **95% CI Lower** | **95% CI Upper** | ***p*** |
| --- | --- | --- | --- | --- | --- | --- |
| C_t_ 🡪 C_t+1_ | 0.198 | 0.00553 | 35.90 | 0.188 | 0.209 | < 0.0001 |
| S_t_ 🡪 C_t+1_ | 0.000398 | 0.00128 | 0.311 | -0.00211 | 0.00290 | 0.7555 |
| C_t_ 🡪 S_t+1_ | -0.00635 | 0.0175 | -0.362 | -0.0407 | 0.0280 | 0.717 |
| S_t_ 🡪 S_t+1_ | 0.165 | 0.00552 | 29.80 | 0.154 | 0.176 | < 0.0001 |

*Note*. C = compliance with physical distancing, S = social support

**Table 5**

*Summarized results of RI-CLPM model for compliance with physical distancing and social satisfaction*

| **Effect** | **Mean estimate** | **Mean SE** | **Z value** | **95% CI Lower** | **95% CI Upper** | ***p*** |
| --- | --- | --- | --- | --- | --- | --- |
| C_t_ 🡪 C_t+1_ | 0.199 | 0.00552 | 36.00 | 0.188 | 0.210 | < 0.0001 |
| S_t_ 🡪 C_t+1_ | 0.0200 | 0.00228 | 8.78 | 0.0155 | 0.0245 | < 0.0001 |
| C_t_ 🡪 S_t+1_ | -0.00712 | 0.00889 | -0.801 | -0.0245 | 0.0103 | 0.4233 |
| S_t_ 🡪 S_t+1_ | 0.142 | 0.00524 | 27.20 | 0.132 | 0.153 | < 0.0001 |

*Note*. C = compliance with physical distancing, S = social satisfaction

**Table 6**

*Summarized results of RI-CLPM model for compliance with staying home measures and social support*

| **Effect** | **Mean estimate** | **Mean SE** | **Z value** | **95% CI Lower** | **95% CI Upper** | ***p*** |
| --- | --- | --- | --- | --- | --- | --- |
| C_t_ 🡪 C_t+1_ | 0.121 | 0.00498 | 24.40 | 0.112 | 0.131 | < 0.001 |
| S_t_ 🡪 C_t+1_ | 0.00426 | 0.000785 | 5.42 | 0.00272 | 0.00579 | < .001 |
| C_t_ 🡪 S_t+1_ | 0.00394 | 0.0294 | 0.134 | -0.0536 | 0.0615 | 0.8933 |
| S_t_ 🡪 S_t+1_ | 0.165 | 0.00552 | 29.80 | 0.154 | 0.176 | < 0.001 |

*Note*. C = compliance with staying home measures, S = social support

**Table 7**

*Summarized results of RI-CLPM model for compliance with staying home measures and social satisfaction*

| **Effect** | **Mean estimate** | **Mean SE** | **Z value** | **95% CI Lower** | **95% CI Upper** | ***p*** |
| --- | --- | --- | --- | --- | --- | --- |
| C_t_ 🡪 C_t+1_ | 0.122 | 0.00498 | 24.40 | 0.112 | 0.131 | < 0.0001 |
| S_t_ 🡪 C_t+1_ | 0.0238 | 0.00143 | 16.70 | 0.0210 | 0.0266 | < 0.0001 |
| C_t_ 🡪 S_t+1_ | 0.00530 | 0.0152 | 0.349 | -0.0245 | 0.0351 | 0.7274 |
| S_t_ 🡪 S_t+1_ | 0.142 | 0.00524 | 27.20 | 0.132 | 0.153 | < 0.0001 |

*Note*. C = compliance with staying home measures, S = social satisfaction

**Table 8**

*Summarized results of RI-CLPM model for compliance with social restriction and social support*

| **Effect** | **Mean estimate** | **Mean SE** | **Z value** | **95% CI Lower** | **95% CI Upper** | ***p*** |
| --- | --- | --- | --- | --- | --- | --- |
| C_t_ 🡪 C_t+1_ | 0.0200 | 0.00782 | 2.56 | 0.00470 | 0.0353 | 0.0104 |
| S_t_ 🡪 C_t+1_ | 0.00706 | 0.00264 | 2.67 | 0.00188 | 0.0122 | 0.0075 |
| C_t_ 🡪 S_t+1_ | -0.00635 | 0.0186 | -0.341 | -0.0429 | 0.0302 | 0.7333 |
| S_t_ 🡪 S_t+1_ | 0.0688 | 0.00888 | 7.75 | 0.0514 | 0.0862 | < 0.0001 |

*Note*. C = compliance with social restriction, S = social support

**Table 9**

*Summarized results of RI-CLPM model for compliance social restriction and social satisfaction*

| **Effect** | **Mean estimate** | **Mean SE** | **Z value** | **95% CI Lower** | **95% CI Upper** | ***p*** |
| --- | --- | --- | --- | --- | --- | --- |
| C_t_ 🡪 C_t+1_ | 0.0201 | 0.00780 | 2.57 | 0.00477 | 0.0353 | 0.0101 |
| S_t_ 🡪 C_t+1_ | 0.00649 | 0.00499 | 1.30 | -0.00329 | 0.0163 | 0.1935 |
| C_t_ 🡪 S_t+1_ | -0.0200 | 0.00970 | -2.06 | -0.0390 | -0.000997 | 0.0391 |
| S_t_ 🡪 S_t+1_ | 0.130 | 0.00836 | 15.60 | 0.114 | 0.147 | < 0.0001 |

*Note*. C = compliance with social restriction, S = social satisfaction

**Table 10**

*Summarized results of RI-CLPM model for compliance with mask wearing and social support*

| **Effect** | **Mean estimate** | **Mean SE** | **Z value** | **95% CI Lower** | **95% CI Upper** | ***p*** |
| --- | --- | --- | --- | --- | --- | --- |
| C_t_ 🡪 C_t+1_ | 0.0293 | 0.00729 | 4.02 | 0.0150 | 0.0436 | 1e-04 |
| S_t_ 🡪 C_t+1_ | 0.0143 | 0.000829 | 17.30 | 0.0127 | 0.0160 | < 0.0001 |
| C_t_ 🡪 S_t+1_ | 0.0294 | 0.0540 | 0.545 | -0.0765 | 0.135 | 0.5859 |
| S_t_ 🡪 S_t+1_ | 0.0679 | 0.00888 | 7.65 | 0.0505 | 0.0853 | < 0.0001 |

*Note*. C = compliance with mask wearing, S = social support

**Table 11**

*Summarized results of RI-CLPM model for compliance with mask wearing and social satisfaction*

| **Effect** | **Mean estimate** | **Mean SE** | **Z value** | **95% CI Lower** | **95% CI Upper** | ***p*** |
| --- | --- | --- | --- | --- | --- | --- |
| C_t_ 🡪 C_t+1_ | 0.0290 | 0.00729 | 3.98 | 0.0147 | 0.0433 | 1e-04 |
| S_t_ 🡪 C_t+1_ | 0.00211 | 0.00158 | 1.34 | -0.000983 | 0.00520 | 0.1811 |
| C_t_ 🡪 S_t+1_ | -0.000467 | 0.0280 | -0.0167 | -0.0553 | 0.0543 | 0.9867 |
| S_t_ 🡪 S_t+1_ | 0.130 | 0.00836 | 15.50 | 0.114 | 0.146 | < 0.0001 |

*Note*. C = compliance with mask wearing, S = social satisfaction

**Table 12**

*Fit measures per model*

| **Compliance variable** | **Social connection variable** | **RMSEA** | **CFI** | **TLI** |
| --- | --- | --- | --- | --- |
| Hygiene measures | Social support | .05 | .99 | .98 |
| Hygiene measures | Social connection | .05 | .95 | .93 |
| Physical distancing | Social support | .05 | .98 | .98 |
| Physical distancing | Social connection | .06 | .92 | .90 |
| Staying home measures | Social support | .05 | .98 | .98 |
| Staying home measures | Social connection | .05 | .93 | .91 |
| Social restriction | Social support | .07 | .98 | .97 |
| Social restriction | Social connection | .08 | .90 | .84 |
| Mask wearing | Social support | .05 | .99 | .98 |
| Mask wearing | Social connection | .07 | .94 | .90 |
|  |  |  |  |  |

*Note*. RMSEA = root mean squared error of approximation, CFI = comparative fit index, TLI = Tucker-Lewis index

**Supplementary Material 5**

**Figure 5**

*Evolution of social connection over time*


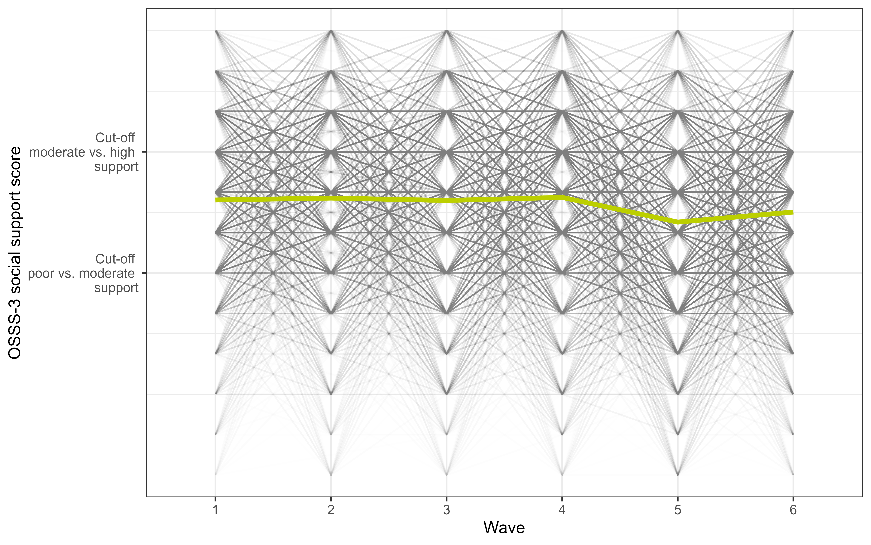

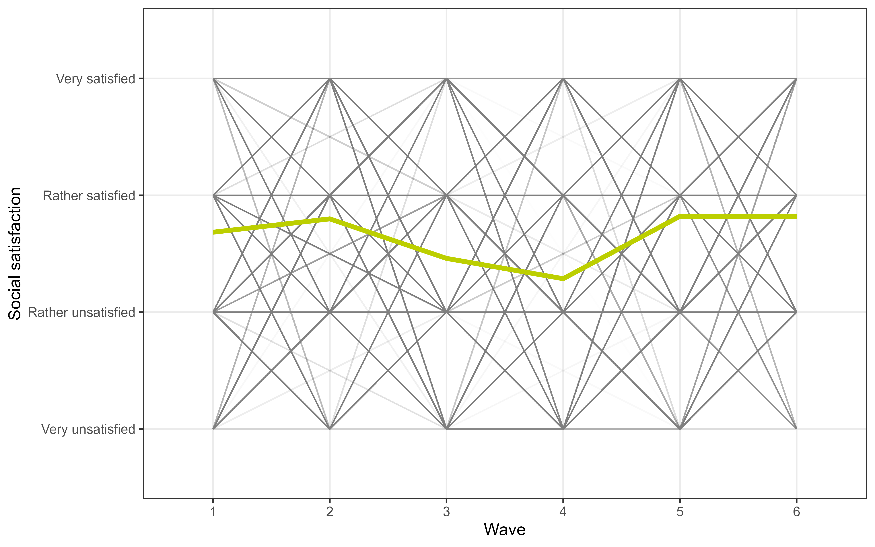


*Note.* Grey lines represent individual trajectories of social connection over time, whereas the green line shows the mean trajectory over participants. Left: evolution of social support OSSS-3 scores over the six survey waves included in the RI-CLPM analyses. Right: evolution of social satisfaction over the six survey waves included in the RI-CLPM analyses.

**Figure 6***Evolution of compliance with preventive measures over time*


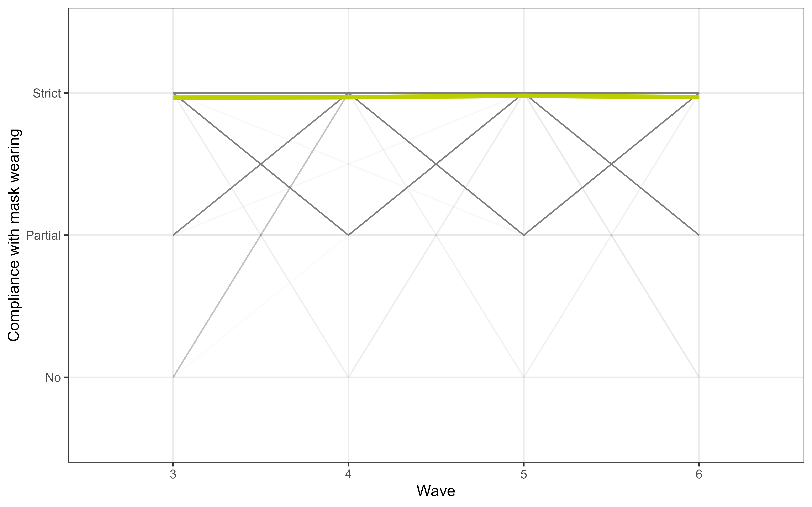

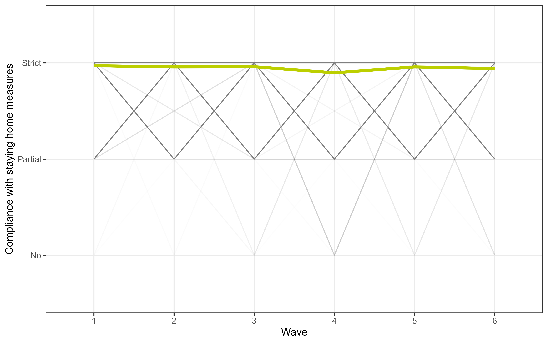

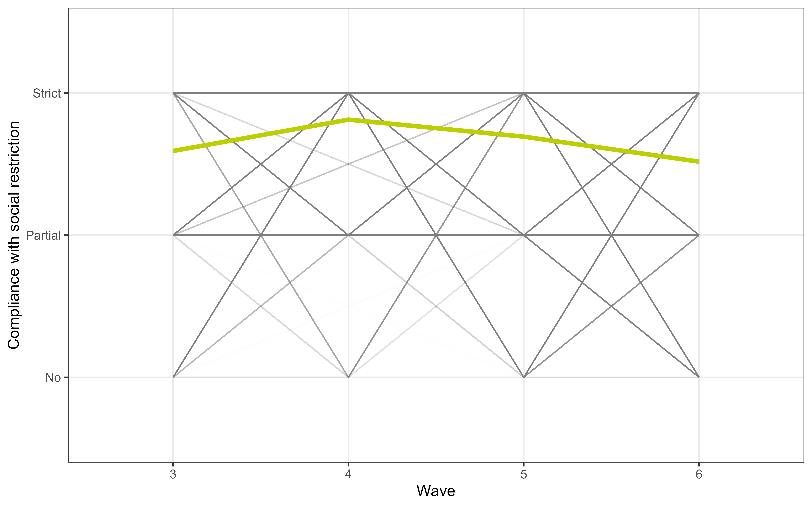

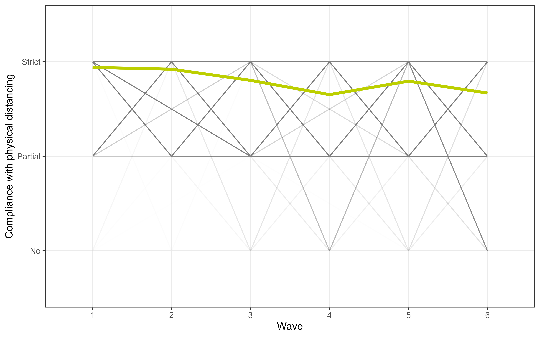

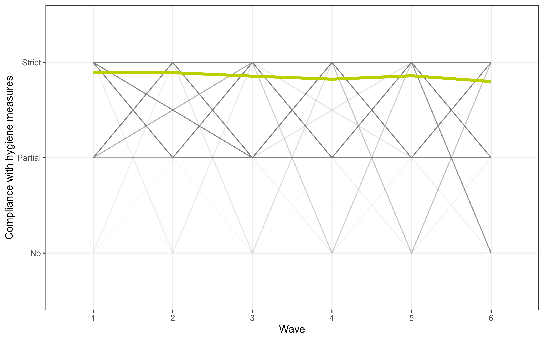


*Note*. Grey lines represent individual trajectories of social connection over time, whereas the green line shows the mean trajectory over participants. Top left: compliance with staying home measures, top middle: compliance with hygiene measures, top right: compliance with physical distancing, bottom left: compliance with mask wearing, bottom right: compliance with social restriction.

**Supplementary Material 6**

**Table 13**

*Summarized results of Bayesian RI-CLPM model for compliance with hygiene measures and social support*

| **Effect** | **Mean estimate** | **Mean SE** | **Mean R-hat** | **95% CrI lower** | **95% CrI upper** | **Excl. zero** |
| --- | --- | --- | --- | --- | --- | --- |
| C_t_ 🡪 C_t+1_ | 0.182 | 0.006 | 1 | 0.189 | 0.211 | Yes |
| S_t_ 🡪 C_t+1_ | 0.0012 | 0.001 | 1 | -0.002 | 0.003 | No |
| C_t_ 🡪 S_t+1_ | 0 | 0.021 | 1 | -0.041 | 0.041 | No |
| S_t_ 🡪 S_t+1_ | 0.165 | 0.006 | 1 | 0.152 | 0.174 | Yes |

*Note*. C = compliance with hygiene measures, S = social support, CrI = credible interval

**Table 14**

*Summarized results of Bayesian RI-CLPM model for compliance with hygiene measures and social satisfaction*

| **Effect** | **Mean estimate** | **Mean SE** | **Mean R-hat** | **95% CrI lower** | **95% CrI upper** | **Excl. zero** |
| --- | --- | --- | --- | --- | --- | --- |
| C_t_ 🡪 C_t+1_ | 0.182 | 0.006 | 1 | 0.189 | 0.211 | Yes |
| S_t_ 🡪 C_t+1_ | 0.0076 | 0.002 | 1 | -0.001 | 0.008 | No |
| C_t_ 🡪 S_t+1_ | -0.0026 | 0.011 | 1 | -0.029 | 0.015 | No |
| S_t_ 🡪 S_t+1_ | 0.142 | 0.005 | 1 | 0.128 | 0.149 | Yes |

*Note*. C = compliance with hygiene measures, S = social satisfaction, CrI = credible interval

**Table 15**

*Summarized results of Bayesian RI-CLPM model for compliance with physical distancing and social support*

| **Effect** | **Mean estimate** | **Mean SE** | **Mean R-hat** | **95% CrI lower** | **95% CrI upper** | **Excl. zero** |
| --- | --- | --- | --- | --- | --- | --- |
| C_t_ 🡪 C_t+1_ | 0.198 | 0.006 | 1 | 0.222 | 0.244 | Yes |
| S_t_ 🡪 C_t+1_ | 0.0002 | 0.001 | 1 | -0.002 | 0.003l | No |
| C_t_ 🡪 S_t+1_ | -0.0064 | 0.018 | 1 | -0.06 | 0.011 | No |
| S_t_ 🡪 S_t+1_ | 0.165 | 0.006 | 1 | 0.153 | 0.175 | Yes |

*Note*. C = compliance with physical distancing, S = social support, CrI = credible interval

**Table 16**

*Summarized results of Bayesian RI-CLPM model for compliance with physical distancing and social satisfaction*

| **Effect** | **Mean estimate** | **Mean SE** | **Mean R-hat** | **95% CrI lower** | **95% CrI upper** | **Excl. zero** |
| --- | --- | --- | --- | --- | --- | --- |
| C_t_ 🡪 C_t+1_ | 0.199 | 0.006 | 1 | 0.223 | 0.245 | Yes |
| S_t_ 🡪 C_t+1_ | 0.02 | 0.002 | 1 | 0.006 | 0.015 | Yes |
| C_t_ 🡪 S_t+1_ | -0.007 | 0.009 | 1 | -0.032 | 0.004 | No |
| S_t_ 🡪 S_t+1_ | 0.142 | 0.005 | 1 | 0.128 | 0.149 | Yes |

*Note*. C = compliance with physical distancing, S = social satisfaction, CrI = credible interval

**Table 17**

*Summarized results of Bayesian RI-CLPM model for compliance with staying home measures and social support*

| **Effect** | **Mean estimate** | **Mean SE** | **Mean R-hat** | **95% CrI lower** | **95% CrI upper** | **Excl. zero** |
| --- | --- | --- | --- | --- | --- | --- |
| C_t_ 🡪 C_t+1_ | 0.121 | 0.005 | 1 | 0.119 | 0.139 | Yes |
| S_t_ 🡪 C_t+1_ | 0.0042 | 0.001 | 1 | -0.001 | 0.002 | No |
| C_t_ 🡪 S_t+1_ | 0.004 | 0.029 | 1 | -0.032 | 0.083 | No |
| S_t_ 🡪 S_t+1_ | 0.165 | 0.006 | 1 | 0.153 | 0.174 | Yes |

*Note*. C = compliance with staying home measures, S = social support, CrI = credible interval

**Table 18**

*Summarized results of Bayesian RI-CLPM model for compliance with staying home measures and social satisfaction*

| **Effect** | **Mean estimate** | **Mean SE** | **Mean R-hat** | **95% CrI lower** | **95% CrI upper** | **Excl. zero** |
| --- | --- | --- | --- | --- | --- | --- |
| C_t_ 🡪 C_t+1_ | 0.121 | 0.005 | 1 | 0.119 | 0.139 | Yes |
| S_t_ 🡪 C_t+1_ | 0.0238 | 0.001 | 1 | 0.005 | 0.01 | Yes |
| C_t_ 🡪 S_t+1_ | 0.0054 | 0.015 | 1 | -0.012 | 0.047 | No |
| S_t_ 🡪 S_t+1_ | 0.142 | 0.005 | 1 | 0.128 | 0.149 | Yes |

*Note*. C = compliance with staying home measures, S = social satisfaction, CrI = credible interval

**Table 19**

*Summarized results of Bayesian RI-CLPM model for compliance with social restriction and social support*

| **Effect** | **Mean estimate** | **Mean SE** | **Mean R-hat** | **95% CrI lower** | **95% CrI upper** | **Excl. zero** |
| --- | --- | --- | --- | --- | --- | --- |
| C_t_ 🡪 C_t+1_ | 0.02 | 0.008 | 1 | 0.005 | 0.035 | Yes |
| S_t_ 🡪 C_t+1_ | 0.00733 | 0.003 | 1 | -0.003 | 0.008 | No |
| C_t_ 🡪 S_t+1_ | -0.00633 | 0.019 | 1 | -0.054 | 0.02 | No |
| S_t_ 🡪 S_t+1_ | 0.0687 | 0.009 | 1 | 0.051 | 0.085 | Yes |

*Note*. C = compliance with social restriction, S = social support, CrI = credible interval

**Table 20**

*Summarized results of Bayesian RI-CLPM model for compliance social restriction and social satisfaction*

| **Effect** | **Mean estimate** | **Mean SE** | **Mean R-hat** | **95% CrI lower** | **95% CrI upper** | **Excl. zero** |
| --- | --- | --- | --- | --- | --- | --- |
| C_t_ 🡪 C_t+1_ | 0.02 | 0.008 | 1 | 0.005 | 0.035 | Yes |
| S_t_ 🡪 C_t+1_ | 0.00633 | 0.005 | 1 | -0.005 | 0.014 | No |
| C_t_ 🡪 S_t+1_ | -0.02 | 0.01 | 1 | -0.048 | -0.01 | Yes |
| S_t_ 🡪 S_t+1_ | 0.13 | 0.008 | 1 | 0.111 | 0.144 | Yes |

*Note*. C = compliance with social restriction, S = social satisfaction, CrI = credible interval

**Table 21**

*Summarized results of Bayesian RI-CLPM model for compliance with mask wearing and social support*

| **Effect** | **Mean estimate** | **Mean SE** | **Mean R-hat** | **95% CrI lower** | **95% CrI upper** | **Excl. zero** |
| --- | --- | --- | --- | --- | --- | --- |
| C_t_ 🡪 C_t+1_ | 0.0297 | 0.007 | 1 | 0.012 | 0.04 | Yes |
| S_t_ 🡪 C_t+1_ | 0.0143 | 0.001 | 1 | 0 | 0.003 | No |
| C_t_ 🡪 S_t+1_ | 0.0293 | 0.053 | 1 | 0.127 | 0.334 | Yes |
| S_t_ 🡪 S_t+1_ | 0.0683 | 0.009 | 1 | 0.05 | 0.084 | Yes |

*Note*. C = compliance with mask wearing, S = social support, CrI = credible interval

**Table 22**

*Summarized results of Bayesian RI-CLPM model for compliance with mask wearing and social satisfaction*

| **Effect** | **Mean estimate** | **Mean SE** | **Mean R-hat** | **95% CrI lower** | **95% CrI upper** | **Excl. zero** |
| --- | --- | --- | --- | --- | --- | --- |
| C_t_ 🡪 C_t+1_ | 0.029 | 0.007 | 1 | 0.012 | 0.04 | Yes |
| S_t_ 🡪 C_t+1_ | 0.002 | 0.002 | 1 | -0.003 | 0.004 | No |
| C_t_ 🡪 S_t+1_ | -0.000333 | 0.027 | 1 | -0.054 | 0.051 | No |
| S_t_ 🡪 S_t+1_ | 0.13 | 0.008 | 1 | 0.111 | 0.144 | Yes |

*Note*. C = compliance with mask wearing, S = social satisfaction, CrI = credible interval

**Table 23**

*Comparison of main RI-CLPM and Bayesian robustness test: effect of social support on subsequent compliance*

| **Social connection variable** | **Compliance measure** | **Main RI-CLPM** | **Bayesian RI-CLPM** | |
| --- | --- | --- | --- | --- |
| *Social connection 🡪 subsequent compliance* | | | | |
| Social support |  |  |  | |
|  | Hygiene measures | / | / | |
|  | Physical distancing | / | / | |
|  | Staying home measures | + | / | |
|  | Social restriction | + | / | |
|  | Mask wearing | + | / | |
| Social satisfaction | Hygiene measures | + | / | |
|  | Physical distancing | + | + | |
|  | Staying home measures | + | + | |
|  | Social restriction | / | / | |
|  | Mask wearing | / | / | |
| *Compliance 🡪 subsequent social connection* | | | | |
| Social support |  |  |  |  |
|  | Hygiene measures | / | | / |
|  | Physical distancing | / | | / |
|  | Staying home measures | / | | / |
|  | Social restriction | / | | / |
|  | Mask wearing | / | | + |
| Social satisfaction | Hygiene measures | / | | / |
|  | Physical distancing | / | | / |
|  | Staying home measures | / | | / |
|  | Social restriction | - | | - |
|  | Mask wearing | / | | / |

*Note.* + : significant/substantial positive effect, - : significant/substantial negative effect, / : not significant or credible interval does not exclude zero

**Supplementary Material 7**

*Education*

The main RI-CLPM analyses were repeated separately for those with and those without tertiary education. This shows a few nuances to the main results, which are also displayed in Figure X. First, when looking at the cross-lagged effects of social connection on subsequent compliance, while most effects found in the overall sample remain present in both groups, the positive effect of social support on subsequent compliance with social restriction was only observed in those without tertiary education (β = 0.005, *p* < .001). Additionally, the effect of social support on subsequent compliance with staying home measures was positive for those with tertiary education (β = 0.007, *p* < .001), but negative for those without (β = -0.005, *p* < .001). While no significant cross-lagged effects of social support on subsequent compliance were found in the overall sample, there were significant positive effects on compliance with hygiene measures specifically for those with tertiary education (β = 0.003, *p* = .021) and on physical distancing for those without tertiary education (β = 0.011, *p* < .001). While the cross-lagged effect of social satisfaction on compliance with mask wearing was not significant in the overall sample, there were opposite, significant effects when taking education into account. More specifically, this particular effect was positive for those with tertiary education (β = 0.008, *p* < .001), but negative for those without tertiary education (β = -0.007, *p* = .034). Second, as was the case in the overall sample, most cross-lagged effects of compliance on subsequent social connection were not significant. However, the negative effect of compliance with social restriction on subsequent social satisfaction was found only in those with tertiary education (β = -0.023, *p* = .037), but not in those without tertiary education (*p* = .564).

**Figure 7***Stratified RI-CLPM results in those with tertiary education*


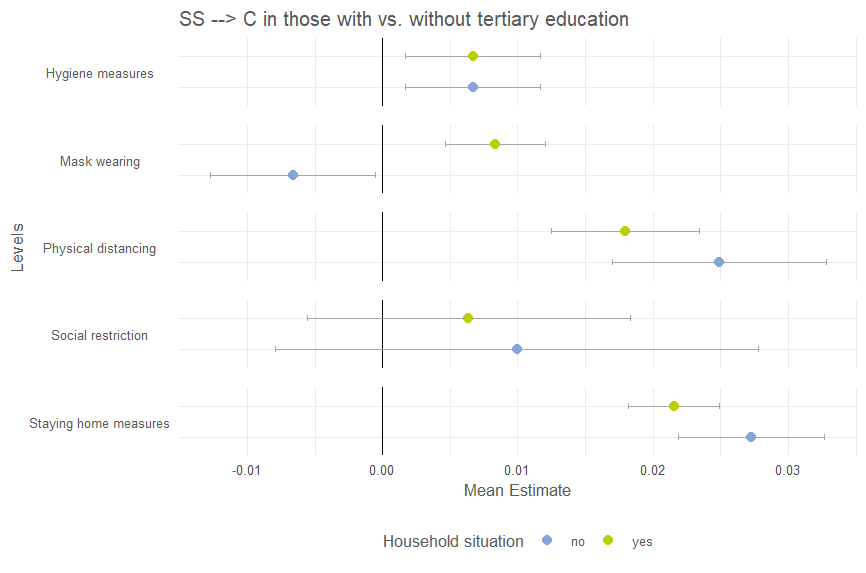

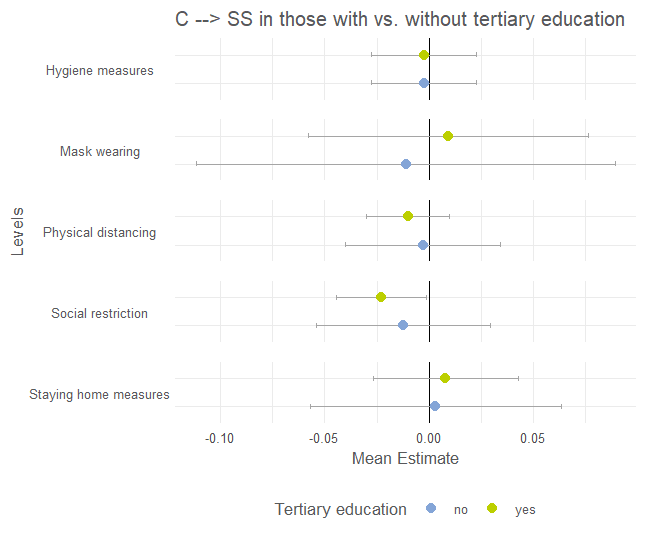

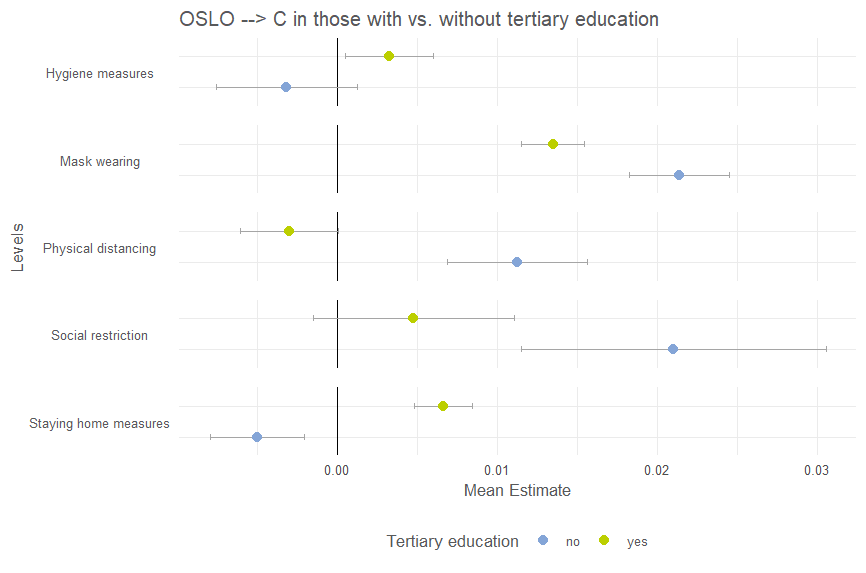

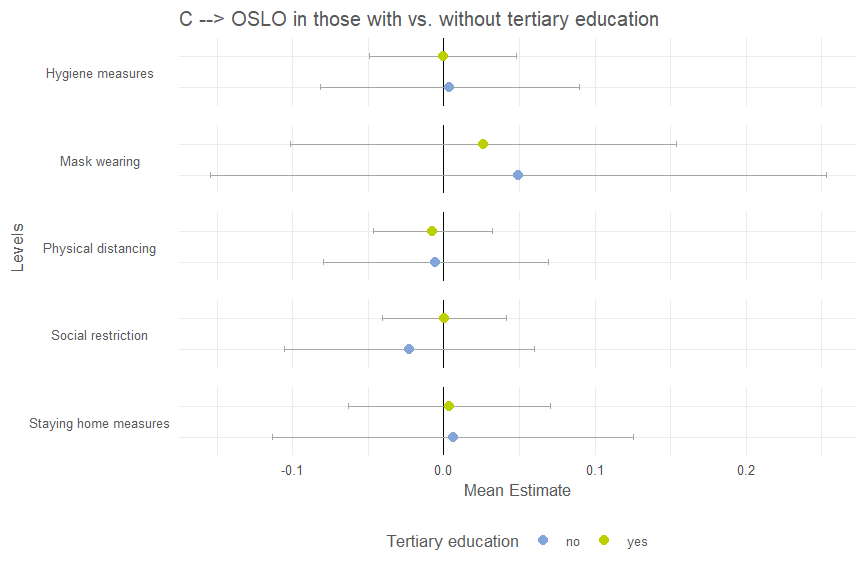

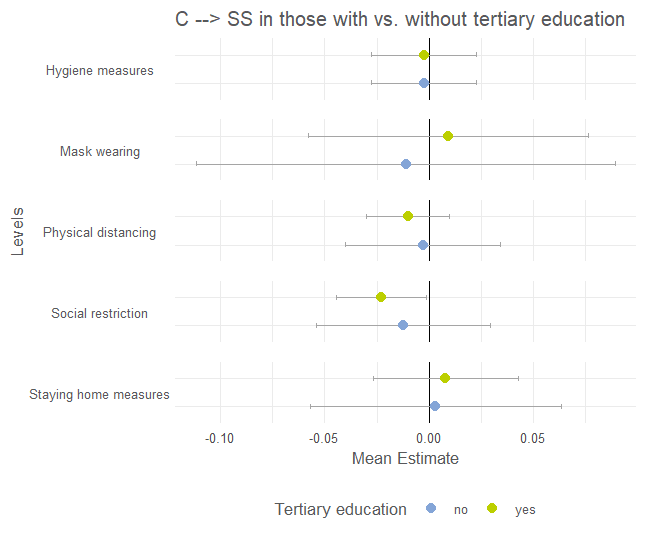

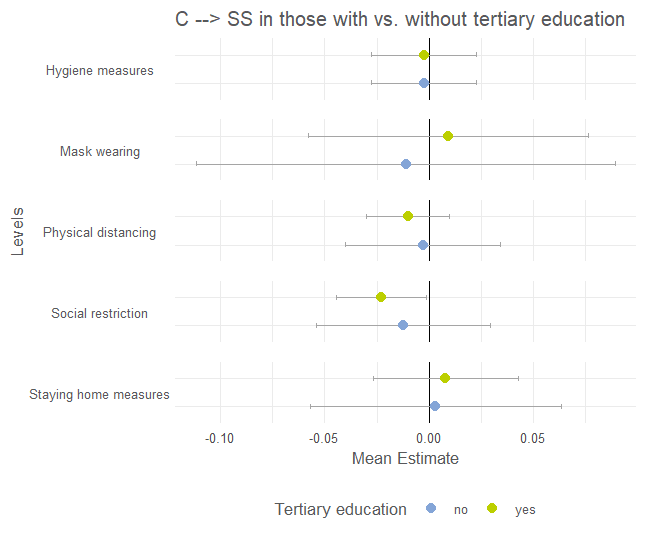


*Note.* C = compliance, SS = social satisfaction, OSLO = social support (Oslo social support scale)

*Sex*

The RI-CLPM analyses were repeated separately for men and women. This shows a few nuances to the main results that are shown in Figure X2. First, for the effects of social connection on subsequent compliance, most results are in line with the main results. However, the positive effect of social support on subsequent compliance with social restriction was only observed in women (β = 0.011, *p* < .001), but not in men (*p* = .708). Similarly, the positive effect of social support on subsequent compliance with staying home measures was observed only in women (β = 0.010, *p* < .001). For men, this effect was even negative (β = -0.005, *p* < .001). Finally, the positive effect of social satisfaction on subsequent compliance with hygiene measures was only significant in men (β = 0.017, *p* < .001), but not in women (*p* = .861). Additionally, whereas there was no overall significant effect of social satisfaction on subsequent compliance with mask wearing, there was a significant positive effect for men (β = 0.011, *p* < .001), but not women (*p* = .144). Second, the findings regarding the (absent) effects of compliance on subsequent social connection were completely in line with the main findings. The negative effect of compliance with social restriction on subsequent social satisfaction was not found in men (*p* = .375) or women (*p* = .062).

**Figure 8***Stratified RI-CLPM results in men and women*


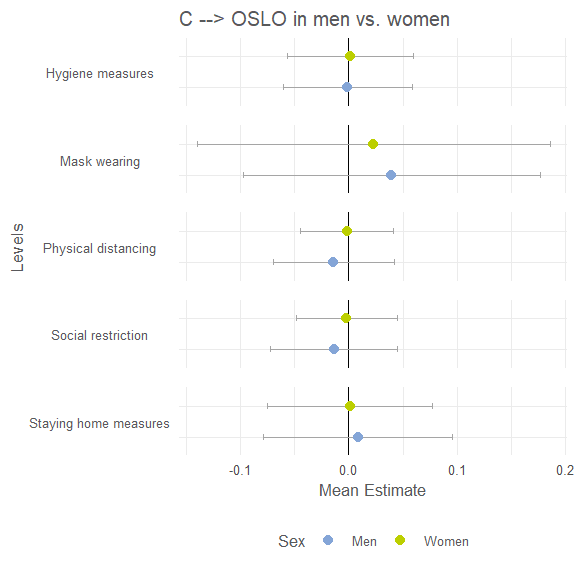

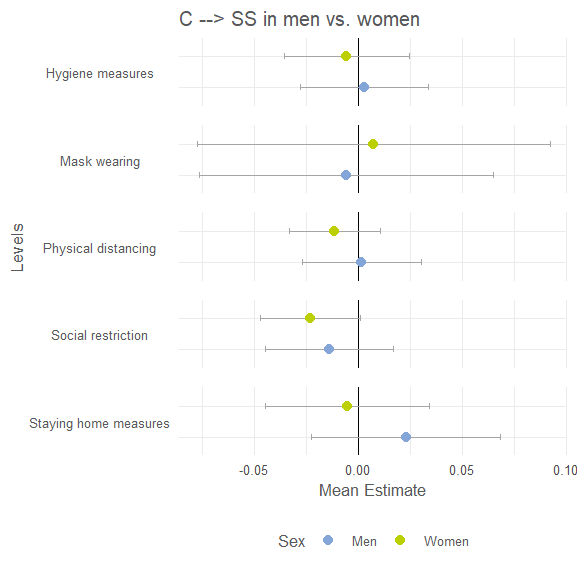

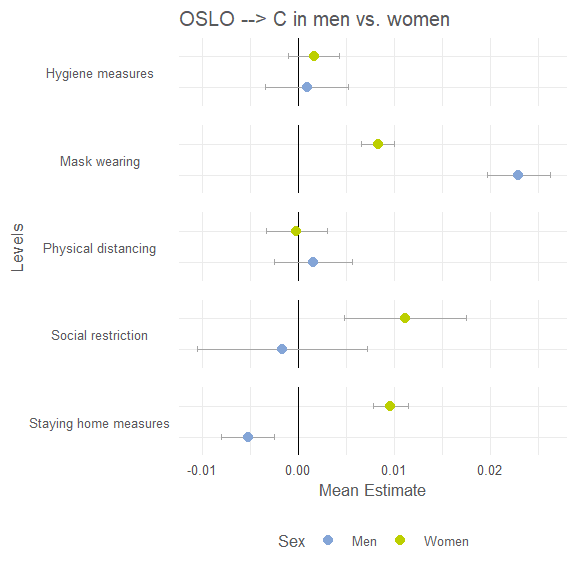

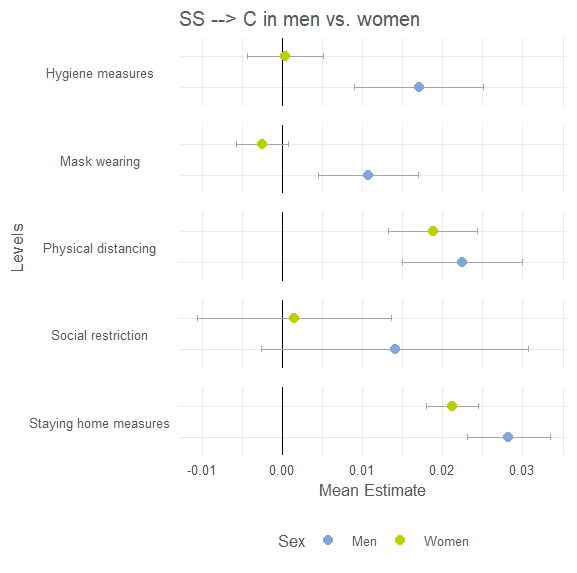


*Note.* C = compliance, SS = social satisfaction, OSLO = social support (Oslo social support scale)

*Household situation*

The RI-CLPM analyses were repeated separately for those living alone and those not living alone. This showed some nuances to the main findings (see Figure X3). First, the effects of social connection on subsequent compliance were mostly in line with the main results, but there were some important nuances and additional effects. The positive effect of social support on subsequent compliance with social restriction was significant only for those living alone (β = 0.020, *p* < .001). The positive effect of social support on subsequent compliance with staying home was only significant for those not living alone (β = 0.006, *p* < .001). The positive effect of social satisfaction on subsequent compliance with physical distancing was only significant for those not living alone (β = 0.025, *p* < .001). Additionally, while there was no significant cross-lagged effect of social support on subsequent compliance with hygiene measures, this effect was positive for those not living alone (β = 0.008, *p* < .001), but negative for those living alone (β = -0.025, *p* < .001). Similarly, the effect of social support on subsequent compliance with physical distancing was positive for those not living alone (β = 0.003, *p* = .034), but negative for those living alone (β = -0.014, *p* < .001). Second, the effects of compliance on subsequent social connection were in line with the main results, showing no significant effects.


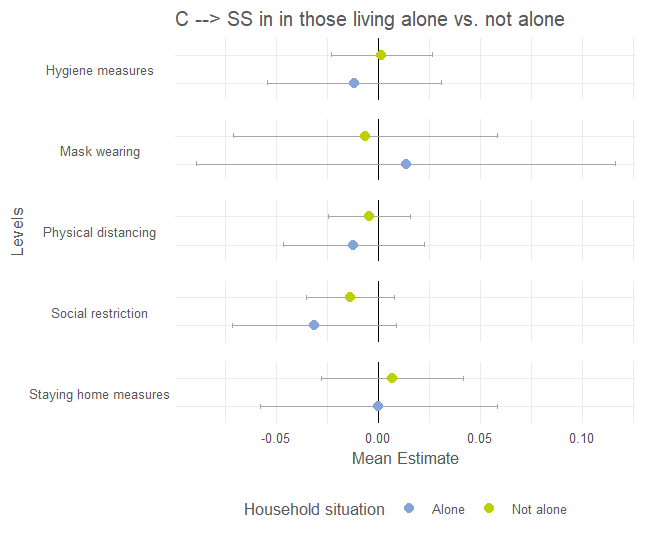
**Figure 9***Stratified RI-CLPM results in those living alone and those not living alone*


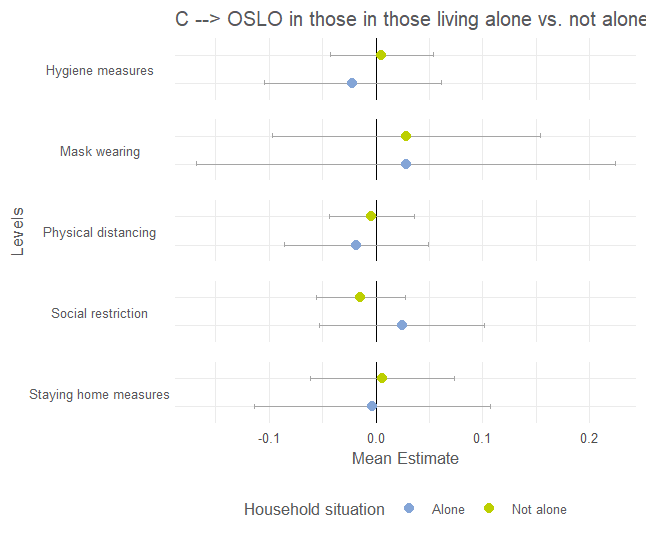

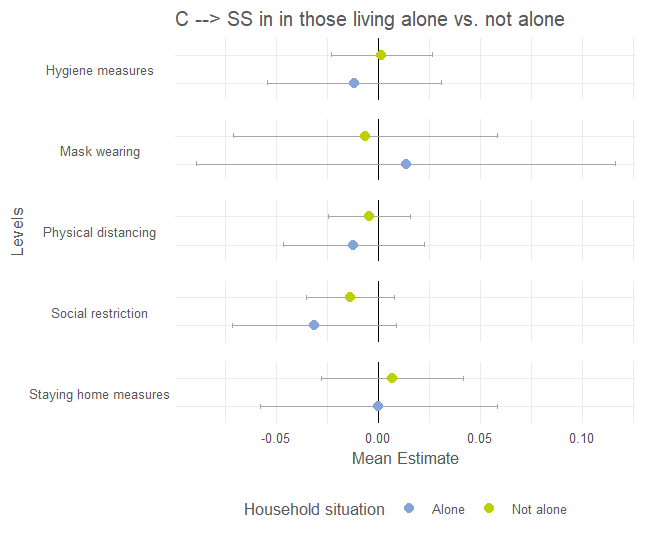

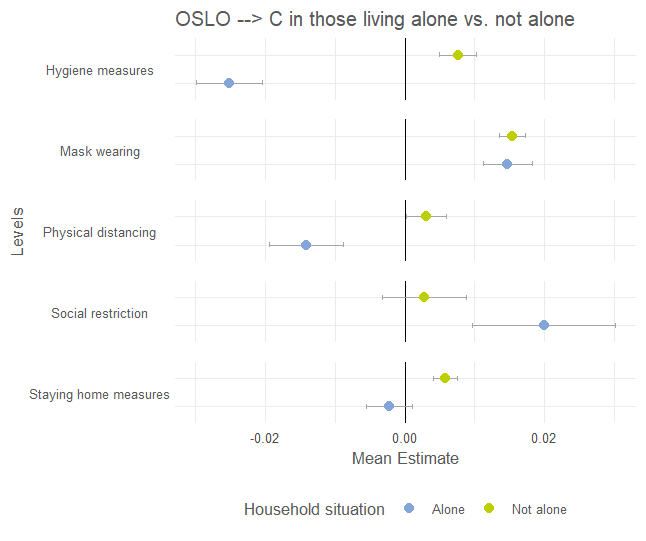

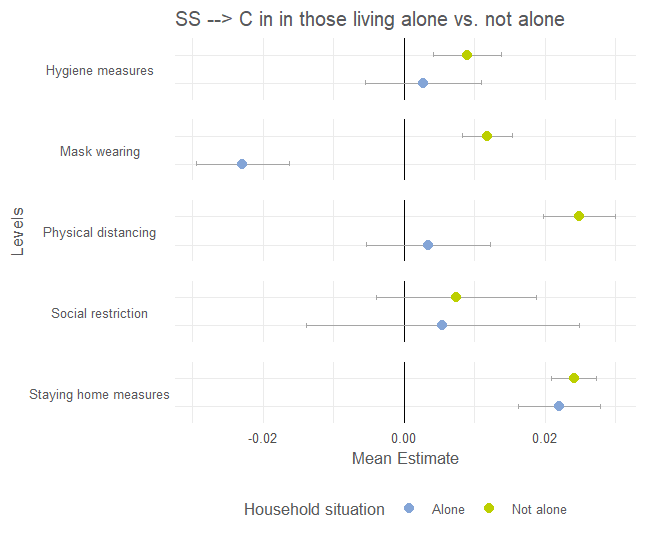


*Note.* C = compliance, SS = social satisfaction, OSLO = social support (Oslo social support scale)

*Age*

Finally, the RI-CLPMs were also performed in separate age groups (see Figure X4). For ease of interpretation, some age categories were collapsed to correspond the following developmental age groups: young adults (18 to 24 years old), adults (25 to 64 years old) and older adults (65 years old and above). First, many effects of social connection on subsequent compliance were in line with the main results, with some nuances and additional effects. The effect of social support on subsequent compliance with mask wearing was significant in all age groups. The general effects of social support on subsequent social restriction and staying home measures were nuanced, in the sense that they were only significant in those aged 65 and older (social restriction: β = 0.020, *p* < .001; staying home measures: β = 0.020, *p* < .001), and, in the case of staying home measures, also in those aged 18 to 24 years old (staying home measures: β = 0.026, *p* < .001). Additionally, whereas the cross-lagged effects for hygiene measures and physical distancing where not significant for the entire sample, they were significant in some age groups. More specifically, there was a significant positive cross-lagged effect of social support on subsequent compliance with hygiene measures in those aged 25 to 64 (β = 0.004, *p* = .013). However, in those aged 18 to 24, this effect was negative (β = -0.021, *p* < .001). There was also a significant positive cross-lagged effect of social support on subsequent compliance with physical distancing in those aged 18 to 24 (β = 0.013, *p* = .042) and 65 and above (β = 0.006, *p* < .001). For social satisfaction, the results were also mainly in line with the overall results, again with some exceptions. The general positive cross-lagged effects for physical distancing and staying home measures were present in all age groups. The effect of social satisfaction on compliance with hygiene measures was found only in those aged 25 to 64 (β = 0.009, *p* < .001), but not in the other age groups. While there was no overall significant cross-lagged effect of social satisfaction on subsequent compliance with social restriction, this effect was significant for those aged 65 years and older (β = 0.022, *p* = .015). For mask-wearing, the age-based analyses yielded mixed results, showing no effect in the age group 25-64 (as in the general analyses), a positive effect in 25 to 64 year-olds (*β* = 0.006, *p* = .003) and a negative effect tin those aged 65 years and older (*β* = -0.007, *p* = .008). Second, the negative cross-lagged effect of compliance with social restriction on subsequent social satisfaction was significant only in the age group of 25 to 64 years (*β* = -0.028, *p* = .013). In line with the main results, all other cross-lagged effects were not significant.

**Figure 10***RI-CLPM results in different age groups*


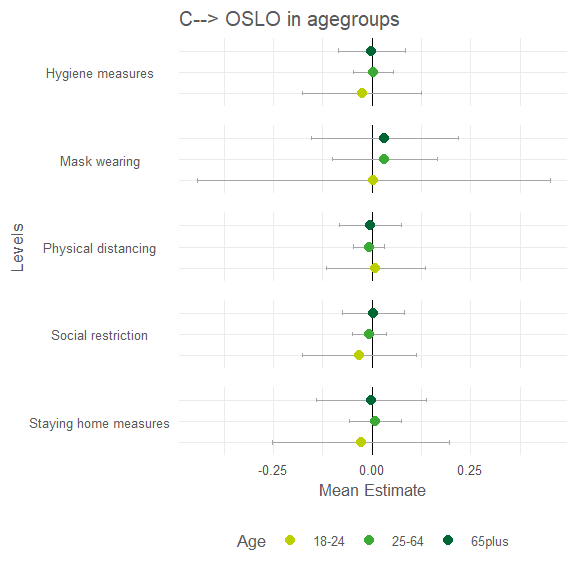

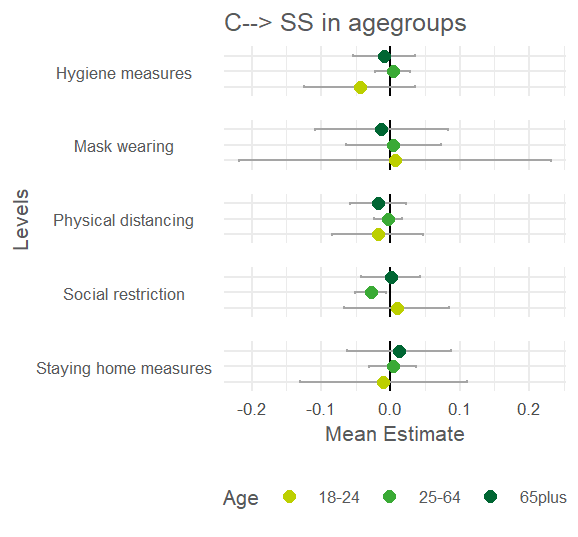

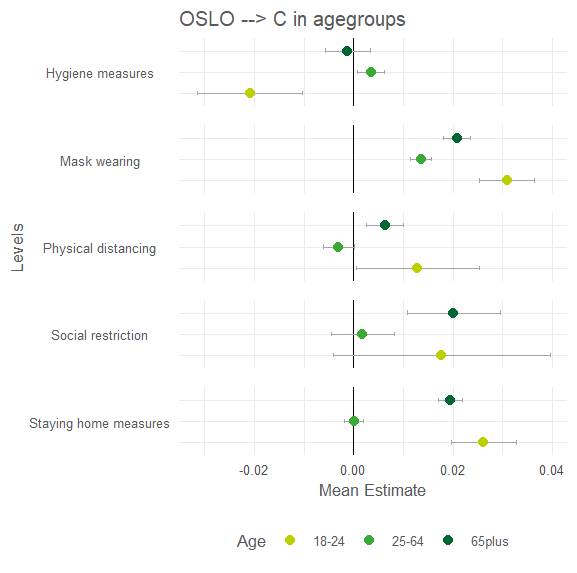

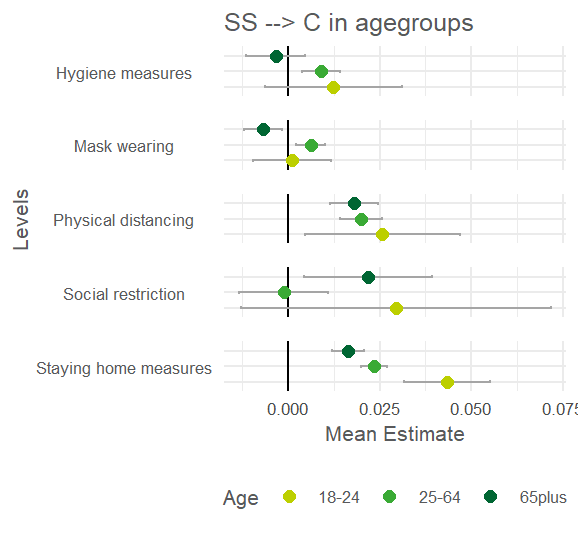


*Note.* C = compliance, SS = social satisfaction, OSLO = social support (Oslo social support scale)
